# Specifications of the ACMG/AMP variant curation guidelines for the analysis of germline *ATM* sequence variants

**DOI:** 10.1101/2024.05.28.24307502

**Published:** 2024-05-29

**Authors:** Marcy E. Richardson, Megan Holdren, Terra Brannan, Miguel de la Hoya, Amanda B. Spurdle, Sean V. Tavtigian, Colin C. Young, Lauren Zec, Susan Hiraki, Michael J. Anderson, Logan C. Walker, Shannon McNulty, Clare Turnbull, Marc Tischkowitz, Katherine Schon, Thomas Slavin, William D. Foulkes, Melissa Cline, Alvaro N. Monteiro, Tina Pesaran, Fergus J. Couch

**Affiliations:** 1. Ambry Genetics, Aliso Viejo, CA, USA; 2. Department of Laboratory Medicine and Pathology, Mayo Clinic, Rochester, MN, USA.; 3. Molecular Oncology Laboratory, Hospital Clínico San Carlos, IdISSC, 28040 Madrid, Spain.; 4. Population Health, QIMR Berghofer Medical Research Institute, Brisbane, QLD 4006, Australia; 5. Department of Oncological Sciences and Huntsman Cancer Institute, University of Utah, Salt Lake City, UT, USA; 6. Natera, Inc, San Carlos, CA, USA; 7. GeneDx, Gaithersburg, MD, USA; 8. Invitae Corporation, San Francisco, CA, USA; 9. Department of Pathology and Biomedical Science, University of Otago, Christchurch, New Zealand; 10. Department of Pathology and Laboratory Medicine, The University of North Carolina at Chapel Hill, Chapel Hill, NC, USA; 11. Division of Genetics and Epidemiology, Institute of Cancer Research, London, UK.; 12. Division of Genetics and Epidemiology, Institute of Cancer Research, London, UK.; 13. City of Hope Comprehensive Cancer Center, Duarte, CA, USA; 14. Departments of Human Genetics, McGill University, Montreal, Quebec, Canada; 15. UC Santa Cruz Genomics Institute, Mail Stop: Genomics, University of California, Santa Cruz, CA, USA; 16. Department of Cancer Epidemiology, H Lee Moffitt Cancer Center & Research Institute, Tampa, FL, USA

## Abstract

The ClinGen Hereditary Breast, Ovarian and Pancreatic Cancer (HBOP) Variant Curation Expert Panel (VCEP) is composed of internationally recognized experts in clinical genetics, molecular biology and variant interpretation. This VCEP made specifications for ACMG/AMP guidelines for the ataxia telangiectasia mutated (*ATM*) gene according to the Food and Drug Administration (FDA)-approved ClinGen protocol. These gene-specific rules for *ATM* were modified from the American College of Medical Genetics and Association for Molecular Pathology (ACMG/AMP) guidelines and were tested against 33 *ATM* variants of various types and classifications in a pilot curation phase. The pilot revealed a majority agreement between the HBOP VCEP classifications and the ClinVar-deposited classifications. Six pilot variants had conflicting interpretations in ClinVar and reevaluation with the VCEP’s *ATM*-specific rules resulted in four that were classified as benign, one as likely pathogenic and one as a variant of uncertain significance (VUS) by the VCEP, improving the certainty of interpretations in the public domain. Overall, 28 the 33 pilot variants were not VUS leading to an 85% classification rate. The ClinGen-approved, modified rules demonstrated value for improved interpretation of variants in *ATM*.

## Introduction

The widespread adoption of low cost, high-throughput, next generation sequencing (NGS)-based multi-gene panel tests has led to a substantial increase in the detection of germline sequence variants. In 2015, in response to this increase, the American College of Medical Genetics and Genomics and the Association for Molecular Pathology (ACMG/AMP) provided a substantial update to their variant interpretation guidelines addressing many of the new challenges for variant interpretation ^1,2^. Because these guidelines are intended for use with any Mendelian disorder, gene- and disease-specific modifications may be needed to develop a tailored approach to variant classification. The process of tailoring variant interpretation guidelines is overseen by the National Institute of Health-funded Clinical Genome Resource (ClinGen) whose mission it is to develop an authoritative, comprehensive, central resource for expert-guided, gene- and variant-level information^3-5^. As part of this ClinGen initiative, the Hereditary Breast, Ovarian and Pancreatic Cancer (HBOP) Variant Curation Expert Panel (VCEP) formed in 2018, with a goal of specifying criteria of the 2015 ACMG/AMP baseline guidelines for clinical classification of variants in *ATM* (MIM 607585), *BARD1* (MIM 601593), *BRIP1* (MIM 605882), *CHEK2* (MIM 604373), *PALB2* (MIM 610355), *RAD51C* (MIM 602774), and *RAD51D* (MIM 602954) (https://clinicalgenome.org/affiliation/50039/). Based on the large number of variants and VUS in ClinVar, the Ataxia Telangiectasia mutated (*ATM*) tumor suppressor gene was selected for initial work of this VCEP.

*ATM* encodes a serine-threonine kinase involved in the cellular response to DNA damage^6^. Heterozygous loss-of-function (LoF) variants in *ATM* are associated with approximately 2-fold increased lifetime risks for breast cancer (MIM#114480) with a penetrance of 20-30%; and a 6.5-fold increased risk for pancreatic cancer ^7-11^. Biallelic pathogenic variants in *ATM* lead to the autosomal recessive disease Ataxia Telangiectasia (A-T) (MIM# 208900)], a severe, early-onset disorder characterized by progressive cerebellar ataxia and ocular telangiectasias ^12^ and increased cancer risk most commonly for leukemia and lymphomas ^13^. Large epidemiological and molecular studies have demonstrated that variants that cause A-T in the biallelic state are also expected to cause increased risk of breast and pancreatic cancer ^14,15^. As such, variants that cause A-T in the biallelic state are also considered by the HBOP VCEP to cause increased risk of breast and pancreatic cancer in the heterozygous state. Given the demonstrated increased risk for autosomal dominant and recessive disease, individuals with likely pathogenic/pathogenic (LP/P) variants in *ATM* may elect to increase cancer surveillance and/or be counseled for family planning. However, there are currently over 7,500 variants of uncertain significance (VUS) deposited to ClinVar, many of which are missense and non-coding variants (https://www.ncbi.nlm.nih.gov/clinvar/?term=atm%5Bgene%5D&redir=gene accessed 3/19/2024).

Therefore, the HBOP VCEP selected *ATM* for development of a validated set of variant classification rule specifications modeled on the baseline 2015 ACMG/AMP guidelines. The gene-specific rules for *ATM* along with application of these rules to curation of a series of *ATM* variants are described herein.

## Methods

### ClinGen HBOP VCEP

The HBOP VCEP formed in 2018 and is comprised of an international team of experts with relevant backgrounds in basic science research including protein functional analysis, clinical genetics, tumor pathology, computational principles, and/or variant interpretation. All members declared conflicts of interest as required by the FDA-approved ClinGen process, including several members who are full time employees at clinical diagnostic laboratories. The HBOP VCEP convened bi-weekly to consider the applicability, weight modifications, and gene-specific nuances of each of the categorical ACMG/AMP guidelines for *ATM*^1^. Initial rules were drafted based on evidence in the literature, internal laboratory data, and expert opinion and approved for pilot phase by ClinGen’s Sequence Variant Interpretation (SVI) group, who oversees this process.

### Pilot Phase

The *ATM*-specific rules were applied in a pilot test of 33 variants comprised of multiple different types (frameshift, nonsense, synonymous, intronic, canonical intronic, missense and structural variants), with different applicable evidence (high frequency variants, rare variants, variants identified in patients with A-T, variants in different functional domains, and variants tested in published functional assays), and/or selected for a variety of clinical assertions in ClinVar. Relevant clinical and allelic data from unpublished sources were solicited from the membership ahead of curation. Two curators independently evaluated variants and compared results. Differences were resolved first by discussion and agreement in a separate biocurator working group, that convened monthly. Differences were then escalated for a secondary review and consensus from the whole HBOP VCEP by vote. If needed, rules were modified or clarified in response to this process.

### Final ATM Rules

Modifications made in response to the pilot study were submitted to the ClinGen SVI for review. The final round of modifications, as recommended by the SVI, were implemented, and resubmitted for approval. Final interpretations for each of the pilot variants were curated into the Variant Curation Interface (VCI) and ultimately deposited to ClinVar. Classifications followed the original five-tier model (Benign, Likely Benign, Variant of Uncertain Significance, Likely Pathogenic and Pathogenic) and evidence combinations with a few modifications that are supported by a Bayesian framework^16^. The most recent *ATM* guidelines can be found on the Criteria Specification Registry and will be updated periodically as the HBOP VCEP continues their work (https://cspec.genome.network/cspec/ui/svi/doc/GN020).

## Results

### Rules not adopted for *ATM* by the HBOP VCEP (PS2, PS4_Moderate, PM1, PM6, PP1, PP2, PP4, PP5, BS2, BS4, BP1, BP3, BP5, BP6)

The HBOP VCEP chose not to adopt numerous ACMG/AMP codes for *ATM* for several reasons (Table 1). First, breast cancer is relatively common and the majority of it is non-hereditary, or sporadic. Second, hereditary and sporadic breast cancer cannot be distinguished from each other at this time. And third, ATM has low penetrance for breast cancer, conveying only two-fold risk which leads to substantial phenocopy and unaffected carriers of pathogenic variants within a family. The codes that were not adopted are detailed below.

#### PS2/PM6: De novo

The observation of a *de novo* variant in the setting of a new disease is evidence towards pathogenicity. The use of *de novo* instances is not informative for *ATM* because breast cancer as a ‘new disease’ cannot be confidently established given the commonness of sporadic breast cancer.

#### PS4_Moderate: Proband Counting

With rare variants where a case control analysis cannot be statistically powered, an approximation called ‘proband counting’ can be used instead. In this method, affected probands can be weighted/counted towards pathogenicity once they reach a certain number that is designed to accommodate the disease and penetrance. It is most useful for pathognomonic gene-disease relationships with high penetrance. Because many genes cause breast cancer predisposition, and because penetrance is low, proband counting does not apply to *ATM*.

#### PP1/BS4: Co-segregation

Segregation of a disease and a variant within the same family is evidence for pathogenicity. However, genes conferring lower risk (Relative Risk = 2) for autosomal dominant conditions, should not be considered for co-segregation analysis because an unrealistic number of pedigrees is needed to obtain a true positive result whilst avoiding a false positive result. For example, in a gene with a Relative Risk of 2, like *ATM*, the probability of obtaining a true positive result for PP1 (as supporting strength) caps at 80% with 40 pedigrees, however that same circumstance also comes with a ~3.5% chance of obtaining a false positive BP4 result ^17^. Regarding the use of BS4 (lack-of-segregation) in families with biallelic A-T is theoretically feasible, however, siblings with the same two variants as the A-T affected proband would be captured under the BP2 code which is a biallelic-unaffected patient.

#### PP4/BS2: Phenotype

A patient who has a phenotype that is highly specific for a disease or an unaffected patient who has not manifested disease can be used in the pathogenic (PP4) or benign (BS2) direction, respectively. However, since hereditary and sporadic breast and pancreatic cancer cannot be distinguished and because the penetrance is low for breast cancer, neither situation can be satisfied for *ATM*.

#### BP5: Variant present in a patient with an alternate mechanism for disease

This rule does not apply, because there are numerous examples of patients carrying both an *ATM* LP/P variant in addition to a second LP/P in other genes whose phenotype is not different than if they were carriers of a single pathogenic variant^18^.

#### PM1: Variant in a functional domain without benign variation

Although ATM has well established functional domains, there are many benign variants described in these domains, based on allele frequency and homozygous occurrences alone (https://gnomad.broadinstitute.org/gene/ENSG00000149311?dataset=gnomad_r2_1 accessed 3/19/2024).

#### PP2 and BP1: Low Rate of Benign Missense Variation

Pathogenic missense variants in *ATM* have been described, and there is not a specified low rate of benign missense variation.

#### BP3: in-frame indels in a repetitive region

There are insufficient data to support the use of in-frame deletions/insertions in a repetitive region without a known function.

#### PP5 and BP6: Reputable Source

These rules regarding reputable sources have been discontinued at the recommendation of the SVI ^19^

### Population Based Rules (BA1, BS1 and PM2_Supporting)

#### BA1 and BS1.

The HBOP VCEP compared parameters for both the autosomal dominant and autosomal recessive conditions ascribed to *ATM* to estimate population allele frequency thresholds using the Whiffen/Ware calculator ^20^; https://cardiodb.org/allelefrequencyapp/).

Because LP/P variants in *ATM* are considered a relatively infrequent cause of hereditary breast cancer the genetic heterogeneity was set to 0.02: in other words, as if 2% of hereditary breast cancer cases are caused by *ATM* LP/P variants. The allelic heterogeneity was conservatively set to 1.0: in other words, assuming that there is only one LP/P variant that causes *ATM*-related breast cancers. Lastly, the penetrance for *ATM* and breast cancer was conservatively set to 0.2 based on data from multiple studies of hereditary breast cancer ^10,11^. Using these parameters, and a prevalence of 1:8 women for breast cancer, the maximum credible allele frequency was 0.625%. Similarly, for A-T the autosomal recessive inheritance was selected along with a prevalence of 1:40,000 ^21-25^. As *ATM* is the only gene that causes A-T, the genetic heterogeneity was set to 1.0 and penetrance was set to 0.90. Using these parameters, the maximum credible allele frequency is 0.527%. Given the conservative parameters put into the calculator and to simplify, the BA1 threshold was set to 0.5%. For BS1, all parameters remained the same except for the extremely conservative allelic heterogeneity value, which was dropped to 0.10, leading to an order-of-magnitude decrease in the maximum credible allele frequency of 0.05%. In applying these frequency codes, statistical models should be considered to account for error related to sample size such as the filtering allele frequency in gnomAD ^26^.

#### PM2.

ClinGen has deviated from the Richards *et al* ACMG/AMP guidelines for PM2 and now recommends that this evidence code be uniformly down weighted to PM2_Supporting (https://www.clinicalgenome.org/site/assets/files/5182/pm2_-_svi_recommendation_-_approved_sept2020.pdf). This recommendation was adopted for rare *ATM* variants. Due to the incomplete penetrance, it is reasonable to expect that unaffected carriers are present in the general population. As such a variant does not need to be absent in the general population to apply PM2_Supporting. For *ATM*, rarity is considered as a general population frequency of ≤0.001% in each subpopulation. Any alteration that exceeds 0.001% in a large general population database but for which there is only one carrier is still considered eligible for PM2_Supporting.

### Loss-of-Function Codes (PVS1 and PVS1(RNA))

#### PVS1

LoF is the mechanism of disease for *ATM* ^22,27,28^. The rules governing the application and appropriate weight of PVS1 are based on the ClinGen SVI recommendations ^29^. There are five variant types that fall under the PVS1 category: nonsense and frameshift alterations; canonical (+/−1,2) splice site alterations (and some last-nucleotide alterations), gross deletions, gross duplications, and initiation codon alterations. Several features influence the weight ascribed to PVS1 including: 1) nonsense-mediated RNA decay (NMD); 2) the impact of an NMD-escaping effect on a critical functional domain 3) the size of the NMD-escaping effect relative to the size of the protein; and 4) gene-specific features.

### *ATM* canonical transcript:

**Figure 1.**
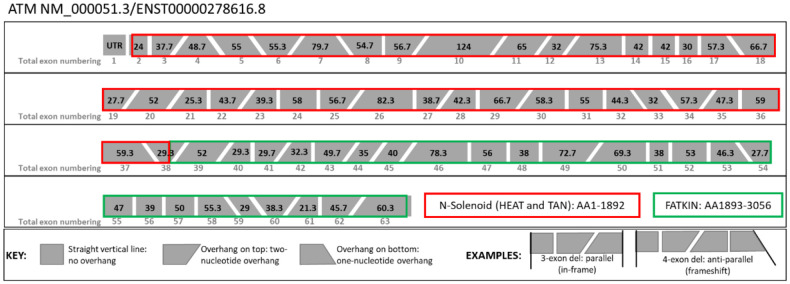
ATM Exon Numbering and Reading Frame. The ATM gene is depicted exon-by-exon. The amino acid size of each exon is depicted within the boxes in black text. The two major functional domains are outlined in red (N-Solenoid, comprised of sub-domains HEAT Repeat and TAN domain) and green outline (FATKIN domain comprised of the FAT and FAT-C sub-domains). Each exon is shaped to indicate the number of overhanging nucleotides at either end which will assist in determining any reading-frame changes from gross deletions or duplications of whole single- or multi-exons. A vertical line indicates a blunt start or end with no overhanging nucleotides. An upper overhang on either side represents a two-nucleotide overhang; A lower overhang represents a single-nucleotide overhang on that side. To use this diagram, a line drawn at the start and end of a deletion or duplication will be either parallel (in-frame event) or non-parallel (frameshift) as in the examples.

The reference transcripts considered by this VCEP are NM_000051.3/ENST00000278616.8. All exons from this transcript are considered constitutive exons without major alternative splice isoforms that could result in a rescue of PVS1-eligible variants ^30-33^. This transcript contains a non-coding first exon (Exon 1) and 62 subsequent coding exons: Exon 2-63 (Figure 1). Of note, *ATM* may be annotated with four non-coding first exons leading to legacy nomenclature references in historical data.

### *ATM* Functional Domains:

*ATM* is comprised of two main functional domains: the N-Solenoid domain and the FATKIN domain. The N-terminal half of the protein is an α-solenoid structure (N-Solenoid) (amino acids 1-1892)^34^ that is able to interact with nucleic acids and various protein partners. The **P**hospho**i**nositide **3-K**inase domain (PI3-K), the **F**ocal **A**dhesion **T**argeting (FAT), and the **F**ocal **A**dhesion **T**argeting **C**arboxyterminal (FATC) collectively comprise the FATKIN domain of ATM (Figure 1). The FATKIN domain is directly responsible for ATM kinase function, which is essential for tumor suppressor activity. Therefore, the FATKIN domain is considered critical for protein function and NMD-escaping variants, including in frame losses and truncations between p.Leu2980 and p.Arg3047, that adversely affect the FATKIN domain are given PVS1 as Very_Strong ^35-38^. The N-Solenoid domain is thought to be important for protein function because there are numerous patients affected with A-T who carry alterations that are known to lead to in-frame losses in the N-Solenoid domain (Supplementary Figure 1) ^30,32,39-49^. However, compared to the FATKIN domain, there are relatively few missense pathogenic mutations (https://www.ncbi.nlm.nih.gov/clinvar/?term=atm%5Bgene%5D&redir=gene accessed 3/19/2024). Because of this, in-frame single- or multi-exon losses impacting the N-Solenoid domain can receive PVS1_Strong.

### PVS1 Eligibility Boundaries:

**Figure 2.**
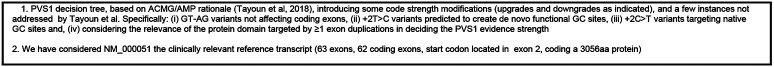

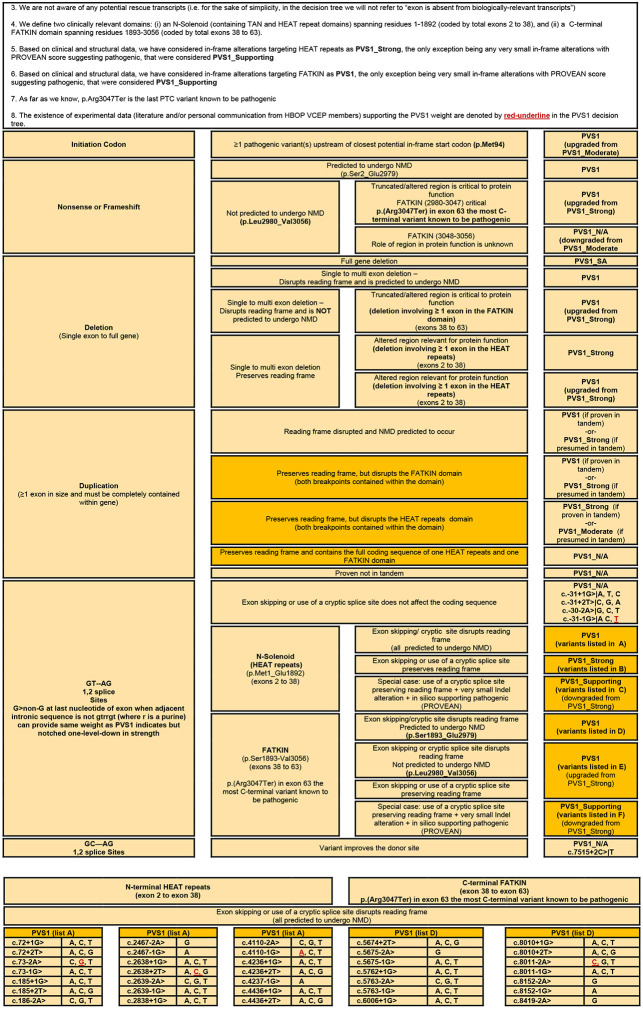

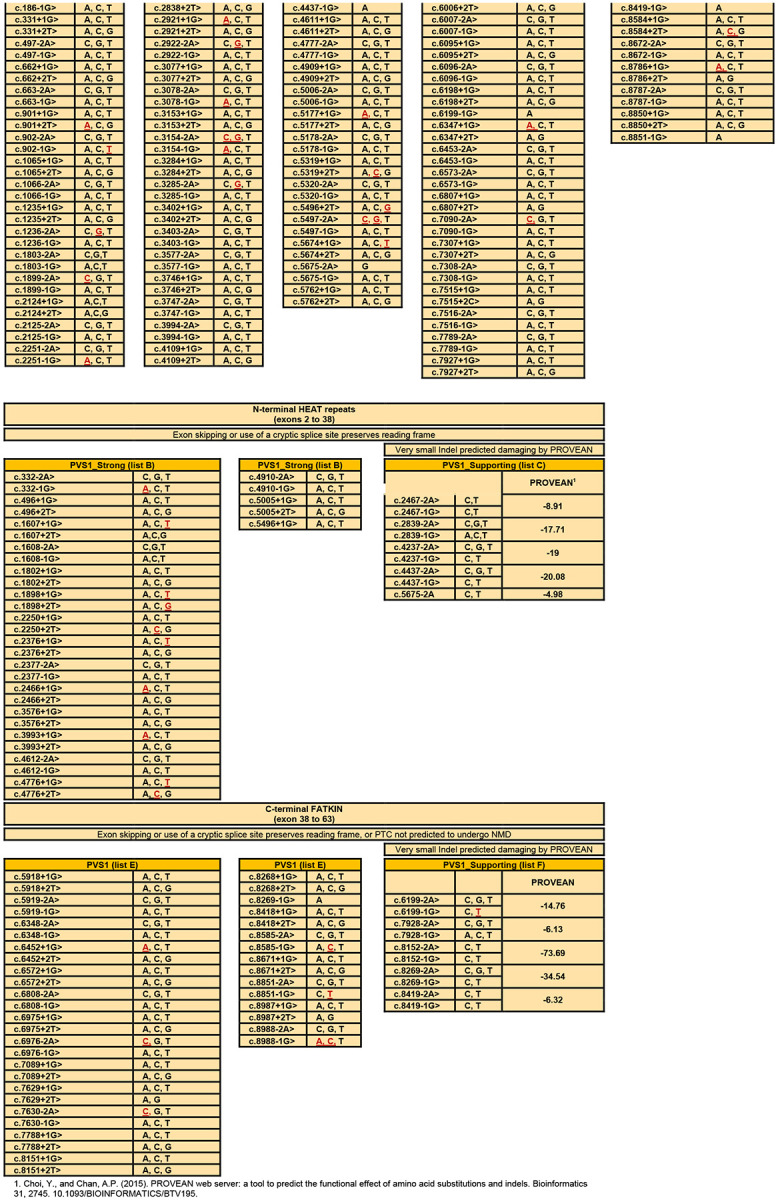
PVS1 Decision Tree for ATM. PVS1 eligible variant types are split into five categories: initiation codon variants, nonsense and frameshift variants, ≥1 exon deletions, ≥1 exon duplications and last NT/canonical splice variants. Considerations related to NMD, N and C terminal boundaries, domain involvement, tandemness, and splice prediction/observation inform the weight that can be afforded to the PVS1 criterion. Small in-frame events were predicted with PROVEAN and scores are provided. Nucleotides in red-underline have splice effects reported in the literature.

Because pathogenic variants in *ATM* cause both A-T in a biallelic state and cancer predisposition in a heterozygous state, this VCEP was able to leverage evidence from A-T cohorts to inform PVS1 boundaries. At the N-terminus, it was determined that variants destroying the initiation codon are ascribed PVS1 as Very_Strong due to the identification of numerous A-T affected individuals harboring p.Met1? variants ^45,47,50-53^. In addition, LoF alterations lying between p.Met1? and the next downstream, in-frame methionine at p.Met94 have also been observed in A-T patients supporting that downstream methionine residues are unable to serve as an alternate start codon that would produce a rescue effect ^54-56^. At the C-terminus, p.Arg3047 is considered the last critical amino acid based on many reports of a nonsense variant at this position in patients with A-T ^30,43,45,47,57-62^. Therefore, LoF alterations impacting codons between p.Met1 and p.Arg3047 are eligible for PVS1 at varying weights according to the PVS1 Decision Tree (Figure 2).

### Gross deletions:

Single-to-multi-exon deletions that are frameshifting and NMD-prone receive PVS1 weight at Very_Strong as per the original 2018 PVS1 guidelines^29^. Alterations producing NMD-escaping transcripts that adversely affect the N-Solenoid receive PVS1_Strong and those adversely affecting the FATKIN domain receive PVS1 as Very_Strong. The HBOP VCEP has made a diagram to assist with discerning the reading frame disruption of gross deletions and duplications (Figure 1).

### Gross duplications:

Single-to-multi-exon duplications that do not involve either the 5’ or 3’ untranslated regions (UTRs) are eligible for PVS1 weight whether they are confirmed or presumed in tandem. PVS1 (as Very_Strong) and PVS1_Strong can be applied for in-frame events confirmed or presumed to disrupt the FATKIN domain, respectively; and PVS1_Strong and PVS1_Moderate can be applied for in-frame events confirmed or presumed to disrupt the N-Solenoid domain, respectively. Care should be taken to ensure that the functional domains are *disrupted* by the duplication which means both the 5’ and 3’ breakpoint of the duplication must be within the same domain. Duplications that have one breakpoint in the N-solenoid domain and one breakpoint in the FATKIN domain do not disrupt either domain and do not receive any PVS1 weight.

### Splice variants:

Canonical splice variants are defined as the +/− 1 and 2 positions in the introns surrounding an exon as well as some alterations at the last nucleotide of the exon. If the sequence does not conform to the consensus U2 donor site of Ggtrrgt (where the capital G is the last-nucleotide position of the exon and where r is any purine) then the impact of a last nucleotide substitution on splicing is expected to be greater. Such alterations are eligible for PVS1 weight but are reduced by one strength level from the corresponding +1,2 baseline weight provided in the PVS1 Decision Tree (Figure 2). Each possible +/−1,2 splice variant is parsed into a PVS1 list (A to F) depending on reading frame and impact on the N-Solenoid or FATKIN domains (Figure 2). Figure 2 was informed by *in silico* score from SpliceAI and/or PROVEAN, in conjunction with published and unpublished splicing data. Of note, there are several variants that receive PVS1_Supporting because they are predicted to make use of an in-frame alternate splice site that preserves the reading frame and leads to a small insertion or deletion that is predicted by PROVEAN to be deleterious (Figure 2, Lists C and F). There are also several candidate variants that do not receive any PVS1 weight because they are +2T>C alterations that do not have a predicted splice impact by SpliceAI. Although rare, +2T>C alterations are known to produce predominantly wildtype transcripts ^63^. There is also one splice site at *ATM* c.7515+2 that is atypical in that it has a native cytosine instead of the consensus thymine. Therefore, a C>T substitution here is predicted to improve the native splice sequence and it receives no PVS1 weight.

### PVS1(RNA):

Any spliceogenic variant, whether canonical, exonic, or deeper intronic, that is confirmed by RNA studies to have a deleterious splice defect can be coded as PVS1(RNA). The application of PVS1(RNA) supplants any other predictive lines of evidence (PVS1 or PP3). Of note, PS3, the code for functional data supporting a pathogenic event, is not used for RNA data because it is reserved for downstream (e.g. protein) functional effects which can be observed in conjunction with an RNA defect and applied in addition to PVS1(RNA). The weight for PVS1(RNA) can be variably ascribed based on curator judgement of the quality and quantitative result of the RNA assay according to recent recommendations by the SVI ^64^. In contrast, RNA functional studies establishing a lack of aberrant splicing can be coded as BP7(RNA). The weight for BP7(RNA) can be variably ascribed from Supporting to Strong based on curator judgment of the quality of the RNA assay.

### Computational/predictive data-driven rules (PS1, PM4, PM5, PP3, BP4, BP7)

#### PS1.

A variant that produces the same protein change as a known pathogenic alteration can be given PS1 towards pathogenicity. This rule may only be applied when a splice defect is ruled out for both the known LP/P alteration and the variant under evaluation by *in silico* splice predictions or RNA evidence. If splicing is a factor for both variants, PS1 can be used as an RNA hotspot and the weight applied is per the ClinGen SVI recommendations (Table 2)^64^.

#### PM4.

In frame deletions and insertions as well as variants disrupting the native stop codon may be eligible for PM4. However, for *ATM*, there are no data available at this time to inform the use of in-frame insertions or deletions. Stop-loss variants in *ATM* are eligible for PM4 due to the identification of numerous A-T patients harboring such pathogenic alterations ^45,56^.

#### PM5.

This rule is ascribed to missense variants at an amino acid residue where another pathogenic missense alteration has been identified. However, amino acid substitutions at a single residue in *ATM* can be pathogenic or benign. Thus, the use of this rule is not recommended. However, this rule has been co-opted as PM5_Supporting to increase the evidence for pathogenicity for LoF alterations being ascribed PVS1 or PVS1(RNA) as Very_Strong. This rule is governed by *ATM’*s lack of alternative splicing events that would produce a functional protein leading to a putative rescue of LoF alterations by splicing the variant out. In this manner, the use of PVS1 and PM5_Supporting will classify all *ATM* LoF variants as likely pathogenic even if they do not meet PM2_Supporting. PVS1/PVS1(RNA)-eligible variants (applied as Very_Strong) that do meet rarity (PM2_Supporting) will be classified as likely pathogenic with the addition of PM5_Supporting.

#### PP3/BP4 Protein.

This VCEP favors the use of the metapredictor REVEL for single nucleotide variation and Provean for small in-frame indels as a single predictor to anticipate the impact of a protein change ^65-67^.A REVEL score ≥0.733 is considered damaging (PP3). And a score ≤0.249 is considered neutral. This threshold is based on the general recommendation and not derived as an ATM-specific threshold at this time^65^. This was further supported by application to prediction of damaging effect in large functional datasets for multiple cancer genes^68^.

#### PP3/BP4 RNA.

The VCEP uses SpliceAI as a sole predictor due to its ability to accurately predict loss of native splice sites and creation of cryptic sites ^69^. This VCEP did not declare gene-specific thresholds for SpliceAI but recommends those set forth by the SVI in applying PP3 to non-canonical splice variants with a SpliceAI score of ≥0.2 and BP4 to variants with a SpliceAI score ≤0.1 ^64^. In the event that RNA data are available and they reflect a substantial variant-specific impact, do not use both PVS1(RNA) and PP3 or BP4. However, in the event that RNA data are available and they reflect no variant-specific impacts, PP3 or BP4 may be applied in conjunction with BP7(RNA) (See Table 3). BP4 may also be used in conjunction with BP7 (see below)^64^.

#### BP7.

This rule was originally intended for synonymous variants, however, the VCEP applied the rule to deep-intronic variants beyond (but not including) +7 at the donor site and −40 at the acceptor site. Per the SVI’s recent guidance, this code is to be applied only when BP4 is met, in which case both BP4 and BP7 would be applied ^64^ (See Table 3). Using these modifications, many synonymous and deep intronic variants can be classified towards benign by applying both BP7 and BP4, in the absence of conflicting data.

### Functional evidence (PS3/BS3)

#### PS3/BS3.

This is applied to protein functional studies or studies that are downstream of RNA effects. For *ATM*, there are multiple well-established functional studies that employ the use of *ATM*-null cell lines to observe the general rescue of radiosensitivity and/or ATM-specific events such as phosphorylation of ATM substrates (Table 4) ^46,70,71^. Because many of the published assays have only a few variants, they contain insufficient known-pathogenic and known-benign controls for Bayesian validation^72^. However, because variant controls in several studies behave as expected in these assays the VCEP has approved a maximum weight of PS3_Moderate and BS3_Moderate for a combination of functional studies that are concordant for a non-functional or functional result, respectively. For non-functional results to be used as PS3_Moderate, both an ATM-specific functional result and a non-specific radiosensitivity functional result should agree. If there is disagreement between results then no weight should be applied towards PS3. If only the ATM-specific-study (e.g. ATM auto- or trans-phosphorylation at specific residues) result is available and reflects non-functional, a maximum weight of PS3_Supporting can be given. However, because a non-functional result from a radiosensitivity assay is not specific to an ATM defect, a non-functional result in a radiosensitivity assay alone does not achieve any PS3 weight. In the benign direction, a neutral result in either an ATM-specific assay or a radiosensitivity assay can be ascribed BS3_Supporting per each. Of note, both PP3/BP4 *in silico* protein predictions and PS3/BP4 protein functional studies can be co-applied.

Note: RNA functional studies reflecting aberrant splicing should be coded as PVS1(RNA) and lack of aberrant splicing as BP7(RNA). Because PS3/BS3 eligible observations measure effects downstream of splicing, it may be appropriate to apply these codes in conjunction with PVS1(RNA)/BP7(RNA).

### Phenotype-related rules (PS4, PM3 and BP2)

#### PS4.

Case-control studies with *ATM* pathogenic variants are expected to yield odds ratio (OR) >2 based on the known increased lifetime breast cancer risks for pathogenic variant carriers ^10,11^. ORs should be statistically significant with a p-value <0.05 and a lower 95% confidence interval >1.5.

#### PM3.

Biallelic pathogenic variants in *ATM* cause A-T. Laboratory studies are available to help rule out differential diagnoses of other ataxia-associated diseases. Of note, A-T can manifest in an atypical fashion, often called variant A-T, that usually presents in childhood with similar features but has a slower progression. The VCEP has created criteria for patients to meet a ‘confident’ or ‘consistent’ *ATM*-associated A-T phenotype with additional weight afforded to those with a ‘confident’ phenotype. There are several considerations in addition to phenotype that need to be reviewed when weighting and applying PM3, including identification of a second *ATM* variant, phase of the second variant, or zygosity, and general population frequency of the variant under consideration. For the application of PM3, points ascribed to multiple probands are additive and the cumulative points can be used as in Table 5 to assign a final weight.

#### BP2.

Each adult (over 18 years of age) without features of A-T that has an *ATM* variant under consideration in the homozygous state, *in trans*, or phase unknown with a LP/P *ATM* variant contributes to BP2 evidence. There are two important considerations in the application of BP2: 1) The source of the data, where a laboratory setting gets stronger weight than a database setting, due increased rigor in the former and risk of a false positive result due to technical issues like allele drop-out in the latter; and 2) Homozygous individuals have a maximum total weight of −2 points (equivalent to BP2_Moderate) no matter how many independent instances there are. This protects against the assumption that a variant is benign when in reality it might be hypomorphic and pathogenic, but an individual may have sub-clinical or very mild features that may be overlooked by a cancer ascertainment bias. The risk of such a phenomenon is reduced in a compound heterozygous state where the other allele is more likely to have typical risks and stronger presentation. One example of very mild homozygous A-T patients (who are affected with dystonia and not cancer) is caused by the founder alteration *ATM* c.6200C>A (p.Ala2067Asp) ^73^. Excepting homozygous cases, multiple cases of biallelic adult patients unaffected by A-T are additive and can be ascribed BP2 weight based on the cumulative points defined in Table 5 up to a maximum weight of BP2_Strong.

### Modified Evidence Code Combinations

The HBOP VCEP adopted the original ACMG-AMP categorical evidence code combinations^1^ with two modifications. To achieve a minimum likely pathogenic classification for PVS1-eligible alterations, the combination of PVS1 plus one additional supporting line of pathogenic evidence is allowed to achieve likely pathogenic. In addition, one strong line of evidence in the benign direction is sufficient to achieve a likely benign classification. Both specific modifications are in line with a Bayesian model of variant interpretation published by the SVI^16^ (Table 6). The use of several code combinations is explicitly permitted or restricted by this VCEP and/or the SVI and these are listed in Table 3.

### Pilot

**Figure 3.**
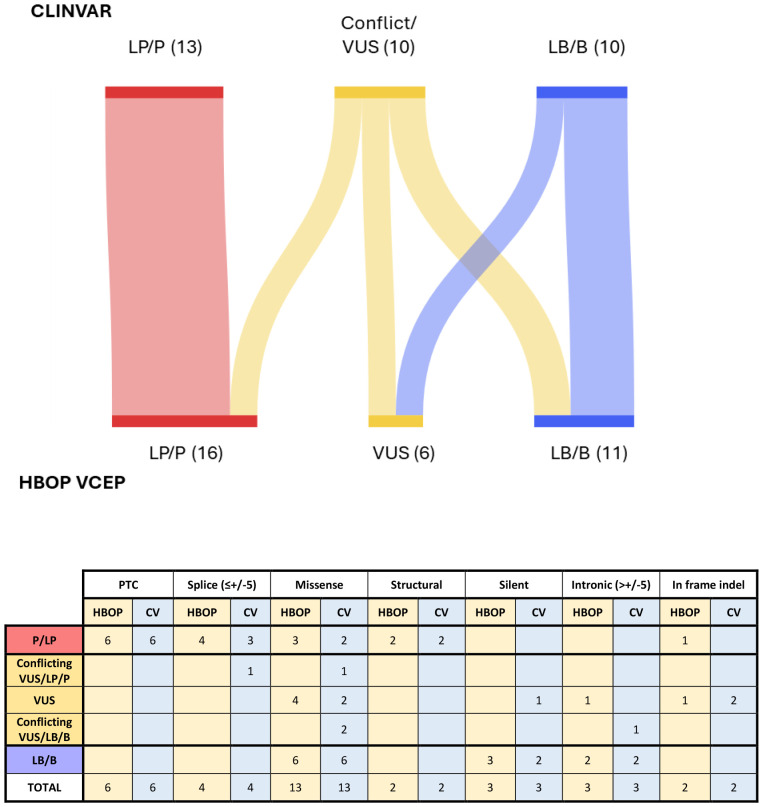
ATM Pilot Variant Categorization. 33 pilot variants are displayed as community classification in ClinVar (left) where VUS/LP/P conflicting interpretation variants and VUS/LB/B conflicting interpretation variants are binned along with consensus VUS as “ClinVar VUS/Conflict”. Interpretation with the HBOP Rules specifications for ATM are on the right. Granular detail of the type of conflict and the type of variant are presented in the table. PTC: Premature Termination Codon; CV: ClinVar

Biocurators evaluated 33 variants of varying type and ClinVar classification in a pilot study. Clinical data were collected regarding co-occurrence data from participating clinical diagnostic laboratories and disseminated in a deidentified fashion to the biocurators. Each variant was reviewed independently by two biocurators who applied lines of evidence for a final classification. Evidence codes and classifications were compared among the biocurator group and reviewed by the HBOP VCEP. Evidence codes and classifications approved by the VCEP were submitted for SVI approval and ultimately deposited to ClinVar. The pilot curation set consisted of 10 non-splicing PVS1-eligible alterations (of a variety of variant types); 13 missense alterations (including one generated by an indel); 7 intronic variants; and 3 synonymous variants. Of these 9 had a consensus B/LB classification in ClinVar, 13 had a consensus P/LP classification in ClinVar; 6 had conflicting interpretations, and 5 were considered a VUS. After developing and applying the VCEP rules, the final classifications achieved were 9 benign variants, 2 likely benign variants, 4 likely pathogenic variants, 12 pathogenic variants, and 6 variants of uncertain significance (Figure 3, Table 7).

Among the variants considered (likely) benign in ClinVar (n=9), the VCEP classified six as (likely) benign and three as VUS (*ATM* c.5556_5557delinsGA (p.Asp1853Asn), *ATM* c.7919C>G (p.Thr2640Ser) and *ATM* c.331+7G>A). Among the variants that were (likely) pathogenic in ClinVar (n=13), the VCEP classified all 13 as (likely) pathogenic. Among 11 variants classified as VUS or conflicting in ClinVar, the VCEP classified five as (likely) benign (four due to application of BA1 or BS1, and one due to the combination of BP4 and BP7), three as (likely) pathogenic (two with PM3_Strong or PM3_Very Strong; and one with the application of PVS1), and three as VUS due to limited evidence (Figure 3, Table 7). The final classifications asserted by the VCEP were submitted to the ClinGen VCI and deposited in ClinVar.

## Discussion

The routine employment of Next-Generation Sequencing represents major advancement in the detection of pathogenic variants in hereditary cancer genes. However, a concomitant and seemingly exponential increase in the detection of variants of uncertain significance is an unfortunate discomfort for many patients and care providers. While it is not possible to resolve the classification of all variants, the development of a set of rules to harmonize classifications across diagnostic and research laboratories can decrease uncertainty related to differential classifications within the public domain. The HBOP VCEP was tasked to define such ACMG/AMP guidelines for *ATM* under the FDA-approved ClinGen VCEP process. This body of work describes the decisions made by the VCEP towards that goal with the ultimate benefit of improving patient outcomes.

The Spanish ATM Working Group (SpATM-WG) defined gene-specific ACMG/AMP style rules for *ATM*, with many similar decisions on rules specifications (Supplementary Table 1) ^74^. However, this VCEP also has substantial departures from the SpATM-WG rules that result largely from a more in-depth analysis related to the FDA-approved process that requires ClinGen SVI and HC-CDWG oversight and collaboration related to rules development. For example, this VCEP has justified the up-weighting of the PM3 and BP2 biallelic codes while the SpATM-WG adopted the original SVI-expounded recommendations (https://clinicalgenome.org/site/assets/files/3717/svi_proposal_for_pm3_criterion_-_version_1.pdf). Another difference is the SpATM-WG assignment of PS3 to variants identified in A-T patients who do not have sufficient ATM expression or substrate phosphorylation. The HBOP VCEP considers this a phenotypic line of evidence rather than a functional line of evidence as this result is not necessarily variant-specific, rather a molecular confirmation of the disease-state of the patient. This concept is incorporated into the VCEP interpretation for PM3. Lastly, among other differences, the HBOP VCEP has elected to omit certain codes for A-T patients that SpATM-WG does apply including *de novo* codes PM6 and PS2, co-segregation codes BS4 and PP1, and PS4 proband counting, which this VCEP applies as PM3.

The careful in-depth consideration of each rule has had an impact on ClinVar classified variants leading to a substantial decrease in the conflicting/VUS rate by nearly 50% (ClinVar n=11; VCEP n=6). The improvement of this VUS rate is likely related to three major features: 1) data sharing of otherwise siloed clinical data among participating clinical diagnostic laboratories towards the application of PM3 and BP2 biallelic codes; 2) the establishment of BA1 and BS1 frequency thresholds leading to the increased number of LB/B variants; and 3) the justified increase in weight applied to A-T patients under PM3 leading to the increased number of LP/P variants. The VCEP is performing ongoing curation and further rule modifications taking into consideration any new information that may be forthcoming, including the development of any new ATM functional studies. Using this method, this VCEP aims to further reduce VUS rates and discordance in variant interpretations submitted to ClinVar with the ultimate goal of improving risk assessment and family genetic counseling.

## Supplementary Material

Supplement 1

## Figures and Tables

**TABLE 1.** SUMMARY OF *ATM* -SPECIFIC RULES SPECIFICATIONS

Code	Original application	ATM-modified application
**PVS1**	Null variant in a gene where loss of function is a known mechanism of disease.	Per *ATM* Exon Map (Figure 1) and *ATM* PVS1 Decision Tree (Figure 2) •PVS1_Variable: Predicted splice defect •PVS1_Variable(RNA): Observed splice defect •NOTE: PVS1 and PVS1(RNA) has code combination restrictions: See Table 3
**PS1**	Same amino acid change as a previously established pathogenic variant regardless of nucleotide change	•Protein: This rule may be applied only when a splice defect is ruled out for both alterations either by RNA analysis and/or in silico splice predictions •RNA (use as PS1_Variable) per SVI guidelines: See PS1 table (Table 4)
**PS2**	De novo (paternity confirmed) in patient with the disease and no family history.	Do not use for AD or AR disease
**PS3**	Well-established in vitro or in vivo functional studies supportive of a damaging effect	•Protein functional studies (Table 5) •PS3_Moderate: A-T (*ATM* null cell line) failure-to-rescue studies (typically target phosphorylation) PLUS confirmatory radiosensitivity assay; • PS3_Supporting: A-T (*ATM* null cell line) rescue study only; •No Weight: radiosensitivty only (non-specific) •RNA functional studies shall be coded as PVS1(RNA) (where RNA is for ‘Observed’)
**PS4**	The prevalence of the variant in affected individuals is significantly increased compared with the prevalence in controls.	•Do not use for proband-counting studies •Case-control studies; p-value ≤.05 AND (Odds ratio, hazard ratio, or relative risk ≥2 OR lower 95% CI ≥1.5).
**PM1**	Located in a mutational hot spot and/or critical and well-established functional domain.	• Do not use: Benign and pathogenic variants are known to occur within the same domains and germline mutational hotspots are not well defined at this time
**PM2**	Absent/rare from controls in an ethnically-matched cohort population sample.	•Variant absent in gnomAD or present in ≤ .001% in all sub-populations •EXCEPTION: under-represented sub-populations with N=1 but frequency >.001% •Not considered a conflicting piece of evidence for variants that otherwise are likely benign/benign •Use as PM2_Supporting (not moderate)
**PM3**	For recessive disorders, detected in trans with a pathogenic variant.	Per A-T PM3 tables (Table 6)
**PM4**	Protein length changes due to in-frame deletions/insertions in a non-repeat region or stop-loss variants.	•Do not use for in frame insertions and deletions as no data are available for this rule at this time •PM4 can be used for stop-loss variants.
**PM5**	Missense change at an amino acid residue where a different missense change determined to be pathogenic has been seen before.	•Do not use for hotspot - Multiple amino acid substitutions at the same residue can be pathogenic or benign and bioinformatic tools cannot yet confidently distinguish them • Apply to frameshifting or truncating variants as PM5_supporting for variants with premature termination codons upstream of p.Arg3047 which are expected to be more severe than the most C-terminal pathogenic varint p.Arg3047*
**PM6**	Confirmed de novo without confirmation of paternity and maternity.	Do not use for AD or AR disease
**PP1**	Co-segregation with disease in multiple affected family members	• AD Condition: Co-segregation analysis in lower-penetrance genes can lead to false positive results • AR Condition: informative instances of co-segregation in A-T families are too rare to be formally analyzed at this time, however, this VCEP supports approaching this similarly to the ITGA2B/ITGB3 and Hearing Loss VCEPs who have outlined PP1 criteria for these autosomal recessive disorders
**PP2**	Missense variant in a gene that has a low rate of benign missense variation and where missense variants are a common mechanism of disease.	Do not use: *ATM* does not have a specified low-rate of benign missense variation.
**PP3**	Multiple lines of computational evidence support a deleterious effect on the gene or gene product	•Protein Analysis: Metapredictor REVEL score ≥.733s •RNA: SpliceAI score ≥.2 •Do not use in conjuction with PVS1(RNA) •Use caution in applying the wrong type of computational evidence (protein vs. RNA) towards the cumulative body of evidence for the opposite mechanism.
**PP4**	Phenotype specific for disease with single genetic etiology.	Do not use for AD disorder For AR disorder, see PM3 for specific phenotype considerations (Table 6)
**PP5**	Reputable source recently reports variant as pathogenic but the evidence is not available to the laboratory to perform an independent evaluation	Do not use
**BA1**	Allele frequency is above 5% in Exome Sequencing Project, 1000 Genomes, or ExAC	>0.5% (.005)
**BS1**	Allele frequency is greater than expected for disorder	>.05% (.0005)
**BS2**	Observed in a healthy adult individual for a dominant (heterozygous) disorder with full penetrance expected at an early age.	Do not use: *ATM* has reduced penetrance
**BS3**	Well-established in vitro or in vivo functional studies shows no damaging effect on protein function	•Protein functional studies **(BS3)** **BS3_Moderate (Protein)**: Both radiosensitivity and *ATM*-null cell line rescue (usually phosphorylation of multiple substrates) are normal. Note ‘Moderate’ does not exist in the current ACMG weights for benign but can be considered as two supporting benign lines of evidence towards final classification **BS3_Supporting (Protein):** Either radiosensitivity OR *ATM*-null cell line rescue (usually phosphorylation of multiple substrates) are normal NOTE: BP4 protein predictions may be used in conjunction with BS3 for protein effects •**RNA:** Do not use: See code BP7_Variable(RNA)
**BS4**	Lack of segregation in affected members of a family.	Do not use: *ATM* has reduced penetrance
**BP1**	Missense variant in gene where only LOF causes disease	Do not use: *ATM* has known missense pathogenic variation
**BP2**	Observed in trans with a pathogenic variant for a fully penetrant dominant gene/disorder.	• See A-T PM3∣BP2 table (Table 6)
**BP3**	In-frame deletions/insertions in a repetitive region without a known function	Do not use
**BP4**	Multiple lines of computational evidence suggest no impact on gene or gene product	•Protein Analysis: Metapredictor REVEL score ≤.249 •RNA: SpliceAI score ≤.1
**BP5**	Variant found in a case with an alternate molecular basis for disease	Do not use
**BP6**	Reputable source recently reports variant as benign but the evidence is not available to the laboratory to perform an independent evaluation	Do not use
**BP7**	A synonymous (silent) variant for which splicing prediction algorithms predict no impact to the splice consensus sequence nor the creation of a new splice site AND the nucleotide is not highly conserved.	BP7: Synonymous and deep intronic •Can be used for deep intronic variants beyond (but not including) +7 (donor) and −40 (acceptor) •May also apply BP4 to achieve Likely Benign •Is not considered a conflicting piece of evidence against a body of evidence supporting a pathogenic splice defect BP7_Variable(RNA): RNA functional studies •Lack of aberrant splice defect: Please see PVS1_Variable(RNA) section (above) for guidance on baseline weights and modifications of weight based on quality for RNA assays •NOTE: BP4 splice predictions may not be used in conjunction with BP7

**TABLE 2.** PS1 CODE WEIGHTS FOR VARIANTS WITH SAME PREDICTED SPLICING EVENT AS KNOWN (LIKELY) PATHOGENIC VARIANT

Variant under assessment (VUA)	Baseline computational/predictive code applicable to VUA	Position of comparison variant relative to VUA	PS1 code applicable to VUA	
			with P comparison variant	with LP comparison variant
Located outside splice donor/acceptor ±1,2 dinucleotide positions	PP3	same nucleotide	PS1	PS1_Moderate
	PP3	within same splice donor/acceptor motif (including at±1,2 positions)	PS1_Moderate	PS1_Supporting
Located at splice donor/acceptor ±1,2 dinucleotide positions	PVS1	within same splice donor/acceptor ±1,2 dinucleotide	PS1_Supporting	N/A
	PVS1	within same splice donor/acceptor region, but outside ±1,2 dinucleotide^a^	PS1_Supporting	PS1_Supporting
	PVS1_Strong, PVS1_Moderate, or PVS1_Supporting	within same splice donor/acceptor ±1,2 dinucleotide	PS1	N/A
	PVS1_Strong, PVS1_Moderate, or PVS1_Supporting	within same splice donor/acceptor region, but outside ±1,2 dinucleotide^a^	PS1_Moderate	PS1_Supporting

Prerequisite for all: the predicted event of the VUA must precisely match the predicted event of the comparison (likely) pathogenic variant (e.g., both predicted to lead to exon skipping, or both to lead to enhanced use of a cryptic splice motif, AND the strength of the prediction for the VUA must be of similar or higher strength than the strength of the prediction for the comparison [likely] pathogenic variant). For an exonic variant, predicted or proven functional effect of missense substitution(s) encoded by the VUA and (likely) pathogenic variant should also be considered before application of this code. Dinucleotide positions refer to donor and acceptor dinucleotides in reference transcript(s) used for curation. Designated donor and acceptor motif ranges should be based on position weight matrices for intron category (see methods). For GT-AG introns these are defined as follows: the donor motif, last 3 bases of the exon and 6 nucleotides of intronic sequence adjacent to the exon; acceptor motif, first base of the exon and 20 nucleotides upstream from the exon boundary. Consider other motif ranges for non-GT-AG introns.

a If relevant, splicing assay data for a pathogenic variant outside a ±1,2 dinucleotide position may be used to update a PVS1 decision tree and hence the applicable PVS1 code for a ±1,2 dinucleotide variant.

**TABLE 3.** RESTRICTIONS ON COMBINING CRITERIA

	PP3	PS3∣BS3	PS1	PVS1	PVS1 (RNA)	BP4	BP7	BP7 (RNA)
PP3		✓	✓	X	X	N/A	N/A	✓
PS3∣BS3	✓		N/A	✓	✓	✓	✓	✓
PS1	✓	N/A		N/A	N/A	N/A	N/A	N/A
PVS1	X	✓	N/A		X	X	X	X
PVS1(RNA)	X	✓	N/A	X		X	X	X
BP4	N/A	✓	N/A	X	X		✓	✓
BP7	X	✓	N/A	X	X	✓		X
BP7(RNA)	✓	N/A	N/A	X	X	✓	X	

N/A: Not applicable because the codes are unrelated

**TABLE 4.** ATM FUNCTIONAL STUDIES

ATM kinase activity			
PMID	18634022	19431188	11805335
DOI / link	10.1002/humu.20805	DOI: 10.1002/humu.21034	10.1073/pnas.012329699
Author	Mitui	Barone	Scott
Year	2009	2009	2002
			
**Assay (general description)**	Stable transfection of ATM-based cDNA constructs in an ATM null cell line; ATM kinase activity assayed by Western Blotting of ATM substrates.	Stable transfection of ATM-based cDNA constructs in an ATM null cell line; ATM kinase activity assayed by Western Blotting of ATM substrates.	Stable transfection of ATM-based cDNA constructs in an ATM null cell line; ATM kinase activity assayed ATM kinase activity assayed by Western Blotting of ATM substrates.
**Material used (patient cells, engineered variants, cell lines, animal model, etc.**	ATM null patient LCL cells (AT7LA1-homozygous for a truncating mutation-c.1563_1564delAG); variants introduced by site-directed mutagenesis.	ATM null patient LCLs (patient 118-3 with two truncating mutations, c.796_797insGATT and c.2921+1G>A) ; variants introduced by site-directed mutagenesis.	ATM null patient LCLs (AT1ABR); variants introduced by site-directed mutagenesis.
**Readout type (qualitative/quantitative)**	qualitative- bands on a gel	quantitative- Western blot images scanned using a densitometer	qualitative-band on a gel
**Readout description**	phosphorylation status of ATM-S1981 and SMC1 (at S957 or S966) tested by Western Blot 1 hr after cells were irradiated with 2 or 10 gy. Cells were treated with CdCl2 to induce ATM expression from a metallothionine promoter II.	Phosphorylation status of SMC1 (Ser-966); NBS1 (Ser-343); CHK2 (Thr-68); p53 (Ser-15); ATM serine 1981 tested by Western Blot at multiple timepoints after cells were irradiated with 5 gray (0, 30 mins, 60 mins) or mock irradiated. Cells were treated with zinc chloride to induce ATM expression from a metallothionine promoter II.	Cells were treated with 6 Gy of ionizing radiation and kinase assays were performed 1 hr after radiation. Phosphorylation status of p53-Ser-15 was assayed directly on cell lysates ("in vivo") or by immunoprecipitation of flag-tagged ATM from cell extracts followed by phosphorylation of a p53 substrate (p531-40, "in vitro"). Cells were treated with CdCl2 6 h before irradiation to induce ATM expression from a metallothionein promoter II.
			
**Biological replicates (met/not met)**	uncertain/not described	not met	not met
**Technical replicates (met/not met); description**	uncertain/not described	Y; 3; not sure if biological or technical	uncertain/not described
**Basic positive control (met/not met); description**	met; WT cDNA (pMAT1) and ATM WT LCLs (NAT2)	met; WT cDNA (pTAM2)	met; WT cDNA (pMAT3)
**Basic negative control (met/not met); description**	met; ATM null cells (AT7LA1)	met; empty cDNA (pMEP4)	met; un-induced cells
**Validation controls P/LP (#)**	0; c.5908C>T (p.Q1970*) - unclear if tested in this assay/data not shown	0	5 AT mutants "c.7636del9,p.SRI2564del3; c.8147T>C, p.V2716A; c.8546G>C p.R2849P; c.8599G>C p.G2867R; c.7987delGTT p.V2662del"
**Validation controls B/LB (#)**	0; c.1744T>C(p.F582L) and c.2119T>C (p.S707P)- unclear if tested in this assay/data not shown	0	0
**Statistical analysis (general description)**	uncertain/not described	uncertain/not specified	uncertain/not described
**Threshold for normal readout**	authors label a normal readout as "normal" in table 1. "normal" is based on the output of the WT construct (pMAT1)	WT level of kinase activity (group 1) (relative to WT pTAM2)	not provided, but curator can distinguish as present or absent
**Threshold for abnormal readout**	authors label an abnormal read out as ND (non-detectable); TD, (trace detected) in table 1.	no detectable kinase activity (group 2) or reduced level of kinase activity (group 3), (relative to WT pTAM2). Authors note that ATM Serine 1981 phosphorylation may not be a good indicator of overall ATM kinase activity.	not provided, but curator can distinguish as present or absent
			
**Approved assay (y/n)**	Y	Y	Y
**Proposed strength**	**PS3_supporting** **BS3_Supporting**	**PS3_Supporting;** **BS3_supporting**	**PS3_Supporting** **BS3_supporting**
			
ATM radiosensitivity			
PMID	18634022	11805335	11805335
DOI / link	10.1002/humu.20805	10.1073/pnas.012329699	10.1073/pnas.012329699
Author	Mitui	Scott	Scott
Year	2009	2002	2002
			
**Assay (general description)**	Stable expression of cDNA constructs in an ATM null cell line; cellular radiosensitivity was assayed as a read-out for ATM function.	Stable transfection of ATM-based cDNA constructs in an ATM null cell line; cellular radiosensitivity was assayed as a read-out for ATM function.	Stable transfection of ATM-based cDNA constructs in an ATM null cell line; cellular radiosensitivity was assayed as a read-out for ATM function.
**Material used (patient cells, engineered variants, cell lines, animal model, etc.**	ATM null patient LCL cells (AT7LA1-homozygous for a truncating mutation-c.1563_1564delAG); variants introduced by site-directed mutagenesis.	ATM null patient LCLs (AT1ABR); variants introduced by site-directed mutagenesis.	ATM null patient LCLs (AT1ABR); variants introduced by site-directed mutagenesis.
**Readout type (qualitative/quantitative)**	quantitative	quantitative	quantitat ive
**Readout description**	% survival fraction (SF) (determined by MTT staining of viable cell colonies after irradiation with 1 gy ). Cells were treated with CdCl2 to induce ATM expression from a metallothionine promoter II.	Cells were treated with 1 Gy of y-rays and fifty metaphases were analyzed for each sample, and radiation induced chromosome aberrations (ICAs) were determined. Cells were treated with CdCl2 6 h before irradiation to induce ATM expression from a metallothionine promoter II	Cells were treated with 1-4 Gy of y-rays and the number of viable cells was determined daily up to 4 days post irradiation. Cells were treated with CdCl2 6 h before irradiation to induce ATM expression from a metallothionein promoter II.
			
**Biological replicates (met/not met)**	uncertain/not specified	uncertain/not specified	
**Technical replicates (met/not met); description**	uncertain/not specified	uncertain/not specified	3; unclear if biological or technical
**Basic positive control (met/not met); description**	met; WT cDNA (pMAT1) and ATM WT LCLs (NAT9)	met; WT cDNA and WT ATM cells (C3ABR)	met; WT cDNA and WT ATM cells (C3ABR)
**Basic negative control (met/not met); description**	met; ATM null cells (AT7LA1)	met; ATM null cells (AT1ABR)	met; ATM null cells (AT1ABR)
**Validation controls P/LP (#)**	0; c.5908C>T (p.Q1970*) (not clear if tested in this assay/data not shown)	5 AT mutants "c.7636del9,p.SRI2564del3; c.8147T>C, p.V2716A; c.8546G>C p.R2849P; c.8599G>C p.G2867R; c.7987delGTT p.V2662del"	5 AT mutants "c.7636del9,p.SRI2564del3; c.8147T>C, p.V2716A; c.8546G>C p.R2849P; c.8599G>C p.G2867R; c.7987delGTT p.V2662del"
**Validation controls B/LB (#)**	0; c.1744T>C(p.F582L) and c.2119T>C (p.S707P') (not clear if tested in this assay/data not shown)	0	0
**Statistical analysis (general description)**	uncertain/not specified	uncertain/not specified	uncertain/not specified
**Threshold for normal readout**	SF >36% (50.1±13.5%) is considered as radio-normal (Sun et al., 2002).	authors state ~ 1 ICA (<1.5 would work)	not specified, propose % survival <10 at 4 days
**Threshold for abnormal readout**	A SF of <21% (13.1±7.2%) has been determined to be the radiosensitive range (Sun et al., 2002).	2.98–3.20 ICAs per metaphase	not specified, propose % survival >10 at 4 days
			
**Approved assay (y/n)**	Y		
**Proposed strength**	**PS3_moderate** (can only be applied if PS3_supporting from kinase assay is met)/ **BS3_Supporting** (can be added with BS3_supporting from kinase assay) **No weight** should be applied if only radiosensitivity is available **No weight** should be applied if radiosensitivity and kinase assay are conflicting	**PS3_moderate** (can only be applied if **PS3_supporting** from kinase assay is met)/ **BS3_Supporting** (can be added with BS3_supporting from kinase assay) **No weight** should be applied if only radiosensitivity is available **No weight** should be applied if radiosensitivity and kinase assay are conflicting	**PS3_moderate** (can only be applied if **PS3_supporting** from kinase assay is met)/ **BS3_Supporting** (can be added with **BS3_supporting** from kinase assay) **No weight** should be applied if only radiosensitivity is available **No weight** should be applied if radiosensitivity and kinase assay are conflicting

**TABLE 5.** PM3 AND BP2 BIALLELIC CODE STRENGTHS

PM3				
Classification/Zygosity of other variant^1^	Points per unrelated A-T Proband (PM3)			
	Confirmed in *trans*	Phase unknown	Second variant unidentified or VUS	Homozygous
Phenotype *confident*	4	2	1	2
Phenotype *consistent*	2	1	0.5	1

BP2			
	Points per Unaffected Adult (>18yo) Proband (BP2)		
	Confirmed in *trans*	Phase Unknown	Homozygous (max −2.0)
Pathogenic or Likely pathogenic variant in a patient	−4	−2	Laboratory Setting −2 Database Setting −1
^1^May not exceed general population frequency > .01%			
**Do not use observations *in cis***			

Points			
Supporting	Moderate	Strong	Very Strong
**PM3**			
**1**	**2**	**4**	**8**
**BP2**			
**−1**	**−2**	**≤−4.0**	**N/A**		

**Phenotype Details**			
**CONFIDENT PHENOTYPE** (must include Laboratory result) Presence of ≥2 Laboratory results 1-4 (see notes) -OR- Presence of Clinical feature 1a or 1b **AND** presence of Laboratory result 1 or 2 -OR- Presence of Clinical feature 2 or 3 **AND** Laboratory result 1 or 2 **CONSISTENT PHENOTYPE** (does not require laboratory result) Presence of two or more Clinical features of ataxia (1a-1e) -OR- Presence of one Clinical feature 1a or 1b **AND** either Clinical feature 2 or 3			
**Clinical features (Neurological and MRI findings):** Progressive cerebellar ataxia, manifesting as: Progressive truncal/limb ataxia Cerebellar degeneration (atrophy of the frontal and posterior vermis and both hemispheres by MRI). Oculomotor apraxia (inability to follow an object across visual fields) or abnormal ocular saccades (rapid refixation from one object to another). Choreoathetosis or dystonia (involuntary movements; twisting and repetitive movements, abnormal postures). Peripheral axonal neuropathy OR Anterior horn cell neuronopathy Oculocutaneous telangiectasia of the conjunctivae, ears, or face. Immunodeficiency (often frequent infections) and/or leukemia/lymphoma.			
**Laboratory Results:** ATM protein levels ≤ 15% of controls in patient fibroblast or lymphoblastoid cell lines. If ATM protein levels are slightly greater than 15%, the ATM kinase activity must be shown to be "negative or low or residual" (see notes). Elevated serum alpha-fetoprotein (AFP) levels >65ug/L in a patient ≥ 2 years old. Increased sensitivity to ionizing radiation in patient fibroblast or lymphoblastoid cell lines. Presence of a 7;14 chromosomal translocation in patient peripheral blood cells (≥ 5% of cells).			
**Notes:** ATM protein levels ≤15% of control levels show >95% sensitivity and >98% specificity for diagnosing (A-T). Protein levels >15% may arise due to a missense variant, a leaky splicing variant, a variant resulting in a kinase-dead protein (where protein levels may not be affected), or a diagnosis other than A-T. When assigning case report criteria based solely on laboratory results (i.e., presence of TWO or more of laboratory results 1-4), there is a greater likelihood that the most specific laboratory results #1 and #2 will be available, and that there will be some clinical indication that the individual(s) has A-T. When assessing homozygous or *in trans* variants (with a likely pathogenic or pathogenic ATM variant) for possible downgrade in an unaffected individual, the individual should be 18 years or older with no evidence of A-T.			

**TABLE 6.** RULES FOR COMBINING CRITERIA

PATHOGENIC CRITERIA
**Pathogenic** 1 Very Strong (PVS1, PVS1(RNA) PM3_VeryStrong) AND ≥1 Strong (PS1-PS4, PM3_Strong, PP1_Strong) OR ≥2 Moderate (PM1-PM6, PP4_Moderate, PP1_Moderate) OR 1 Moderate (PM1-PM6, PP4_Moderate, PP1_Moderate) and 1 Supporting (PP1-PP5, PM3_Supporting) OR ≥2 Supporting (PP1-PP5, PM3_Supporting) ≥2 Strong (PS1-PS4, PM3_Strong, PP1_Strong) OR 1 Strong (PS1-PS4, PM3_Strong, PP1_Strong) AND ≥3 Moderate (PM1-PM6, PP4_Moderate, PP1_Moderate) OR 2 Moderate (PM1-PM6, PP4_Moderate, PP1_Moderate) AND ≥2 Supporting (PP1-PP5, PM3_Supporting) OR 1 Moderate (PM1-PM6, PP4_Moderate, PP1_Moderate) AND ≥4 Supporting (PP1-PP5, PM3_Supporting) 1 Moderate (PM1-PM6, PP4_Moderate, PP1_Moderate) AND ≥4 Supporting (PP1-PP5, PM3_Supporting)
**Likely Pathogenic** 1 Very Strong (PM3_VeryStrong) AND 1 Moderate (PP1-PP5, PM3_Supporting) OR 1 Very Strong (PVS1, PM3_VeryStrong) AND 1 Supporting (PP1-PP5, PM3_Supporting) OR 1 Strong (PS1-PS4, PM3_Strong, PP1_Strong) AND 1-2 Moderate (PM1-PM6, PP4_Moderate, PP1_Moderate) OR 1 Strong (PS1-PS4, PM3_Strong, PP1_Strong) AND ≥2 Supporting (PP1-PP5, PM3_Supporting) OR ≥3 Moderate (PM1-PM6, PP4_Moderate, PP1_Moderate) OR 2 Moderate (PM1-PM6, PP4_Moderate, PP1_Moderate) AND ≥2 Supporting (PP1-PP5, PM3_Supporting) OR 1 Moderate (PM1-PM6, PP4_Moderate, PP1_Moderate) AND ≥4 Supporting (PP1-PP5, PM3_Supporting)
BENIGN CRITERIA
**Benign** 1 Stand-Alone (BA1) OR ≥2 Strong (BS1-BS4)
**Likely Benign** 1 Strong OR 1 Strong (BS1-BS4) and 1 Supporting (BP1-BP7, BS3_Supporting, BP7 _Supporting(RNA)) OR ≥2 Supporting (BP1-BP7, BS3_Supporting, BP7_Supporting(RNA))

**TABLE 7.** PILOT VARIANTS RESULTS

Variant Type	Variant Information	ClinVar ID	Allele Registry ID	ClinVar Classification	ClinVar Star	HBOP Final Classification	HBOP Curation Criteria Applied
frameshift indel	NM_000051.4(ATM):c.1122_1123d el (p.Glu376IlefsTer2)	818362	n/a	P/LP	2 star	Pathogenic	PM2_supporting, PVS1, PM3, PM5_supporting
frameshift indel	NM_000051.4(ATM):c.3245_3247d elinsTGAT (p.His1082LeufsTer14)	3033	CA298025	P/LP	2 star	Pathogenic	PVS1, PM2_supporting, PM3_very-strong, PM5_supporting
frameshift indel	NM_000051.4(ATM):c.6997dup	140818	CA345709	P/LP	2 star	Pathogenic	PM2_supporting, PVS1, PM3_strong, PM5_supporting
gross del	NC_000011.10:g.(?_108287594)_(108287721_?)del	453341	n/a	P	1 star	Pathogenic	PVS1, PM2_Supporting, PM5_Supporting
gross dup	NC_000011.9:g.(?_108137888)_(108225611_?)dup	583716	n/a	P	1 star	Likely pathogenic	PVS1_Strong, PM3, PM2_Supporting
in frame indel	NM_000051.4(ATM):c.8578_8580d elTCT(p.Ser2860del)	3018	CA198490	VUS	2 star	Pathogenic	PM3_very-strong, PM2_supporting, PP3
in frame indel	NM_000051.4(ATM):c.1905_1910d el (p.His635_His636del)	141289	CA165011	VUS	2 star	Uncertain significance	PM2_supporting, PP3
missense	NM_000051.4(ATM):c.2614C>T (p.Pro872Ser)	133610	CA157083	B/LB	2 star	Benign	BA1, BP2_strong, BP4
missense	NM_000051.4(ATM):c.3118A>G (p.Met1040Val)	3027	CA151920	B/LB	2 star	Benign	BA1, BP2_strong, BP4
missense	NM_000051.4(ATM):c.6995T>C (p.Leu2332Pro)	133631	CA157165	B/LB	2 star	Benign	BA1, BP4, BP2_strong
missense	NM_000051.4(ATM):c.146C>G (p.Ser49Cys)	3048	CA202190	B/LB	1 star	Benign	BA1, BP2_strong
missense (indel)	NM_000051.4(ATM):c.5556_5557d elinsGA (p.Asp1853Asn)	929198	n/a	B/LB	2 star	Uncertain significance	PM2_supporting, PP3
missense	NM_000051.4(ATM):c.1073A>G (p.Asn358Ser)	127329	CA286708	Conflicting B/LB/VUS	1 star	Benign	BS1, BP2_strong, BP4
missense	NM_000051.4(ATM):c.3925G>A (p.Ala1309Thr)	127377	CA242620	Conflicting B/LB/VUS	1 star	Benign	BS1, BP2_strong, BP4
missense	NM_000051.4(ATM):c.3284G>A (p.Arg1095Lys)	141522	CA165678	Conflicting LP/VUS	1 star	Uncertain significance	PM2_supporting, PVS1_strong
missense	NM_000051.4(ATM):c.7919C>G (p.Thr2640Ser)	231842	CA10579274	LB	1 star	Uncertain significance	PM2_supporting
missense	NM_000051.3(ATM):c.8546G>C (p.Arg2849Pro)	490737	CA382518439	LP	2 star	Likely pathogenic	PS3_moderate, PM2_supporting, PP3, PM3
missense	NM_000051.4(ATM):c.7271T>G (p.Val2424Gly)	3023	CA115930	P/LP	2 star	Pathogenic	PS3_moderate, PS4, PM3_very-strong, PP3, PP1
missense	NM_000051.4(ATM):c.3137T>C (p.Leu1046Pro)	186558	CA195169	VUS	2 star	Likely pathogenic	PM2_supporting, PP3, PM3_strong, PP4
missense	NM_000051.3(ATM):c.8734A>G (p.Arg2912Gly)	133641	CA157198	VUS	2 star	Uncertain significance	PP3, BP2_strong
nonsense (C-term)	NM_000051.4(ATM):c.9139C>T (p.Arg3047Ter)	3029	CA115937	P	2 star	Pathogenic	PS3_supporting, PM3_very-strong, PVS1
nonsense (mid)	NM_000051.4(ATM):c. 1442T>G (p.Leu481Ter)	453367	CA382534080	P/LP	2 star	Pathogenic	PM2_supporting, PM3_strong, PVS1, PM5_supporting
splice	NM_000051.4(ATM):c.2639-17G>T	140763	CA163513	B	2 star	Benign	BA1, BP4, BP2_strong
splice	NM_000051.4(ATM):c. 1066-6T>G	3038	CA151456	Conflicting B/LB/VUS	1 star	Benign	BS1, BP2_strong
splice	NM_000051.4(ATM):c.8268+1G>A	420799	CA16619252	Conflicting LP/VUS	1 star	Likely pathogenic	PVS1, PM2_supporting
splice	NM_000051.4(ATM):c.332-1G>A	231535	CA6264590	LP	2 star	Pathogenic	PM3_strong, PM2_supporting, PVS1_strong
splice	NM_000051.4(ATM):c.1607+1G>T	220555	CA348209	P/LP	2 star	Pathogenic	PM3_very-strong, PVS1_strong, PM2_supporting
splice	NM_000051.4(ATM):c.8585-2A>C	407718	CA16613454	P/LP	2 star	Pathogenic	PVS1, PM2_supporting, PM3_strong
splice	NM_000051.4(ATM):c.331+7G>A	453461	CA658656149	LB	2 star	Uncertain significance	BP4
start loss	NM_000051.4(ATM):c.2T>C (p.Met1?)	187275	CA197209	P/LP	2 star	Pathogenic	PVS1, PM2_supporting, PM3_very-strong, PP4
synonymous	NM_000051.4(ATM):c.1176C>G (p.Gly392=)	142140	CA167509	B	2 star	Benign	BP2_strong, BA1, BP4, BP7
synonymous	NM_000051.4(ATM):c.5544T>C (p.Asp1848=)	184944	CA190548	LB	2 star	Likely benign	BP4, BP7
synonymous	NM_000051.4(ATM):c.8751C>T (p.Gly2917=)	453745	CA6266433	VUS	2 star	Likely benign	BP4, BP7, PM2_supporting

## References

[1] Richards S., Aziz N., Bale S., Bick D., Das S., Gastier-Foster J., Grody W.W., Hegde M., Lyon E., Spector E., (2015). Standards and guidelines for the interpretation of sequence variants: a joint consensus recommendation of the American College of Medical Genetics and Genomics and the Association for Molecular Pathology. Genet Med 17, 405–424. 10.1038/GIM.2015.30. 25741868 PMC4544753

[2] Plon S.E., Eccles D.M., Easton D., Foulkes W.D., Genuardi M., Greenblatt M.S., Hogervorst F.B.L., Hoogerbrugge N., Spurdle A.B., and Tavtigian S. V. (2008). Sequence variant classification and reporting: recommendations for improving the interpretation of cancer susceptibility genetic test results. Hum Mutat 29, 1282–1291. 10.1002/HUMU.20880. 18951446 PMC3075918

[3] Nussbaum R.L., Rehm H.L., and ClinGen (2015). ClinGen and Genetic Testing. N Engl J Med 373, 1377–1378. 10.1056/NEJMc1508700. 26430707

[4] Rehm H.L., Berg J.S., Brooks L.D., Bustamante C.D., Evans J.P., Landrum M.J., Ledbetter D.H., Maglott D.R., Martin C.L., Nussbaum R.L., (2015). ClinGen--the Clinical Genome Resource. N Engl J Med 372, 2235–2242. 10.1056/NEJMSR1406261. 26014595 PMC4474187

[5] Karam R., Pesaran T., and Chao E. (2015). ClinGen and Genetic Testing. N Engl J Med 373, 1376–1377. 10.1056/NEJMc1508700. 26422737

[6] Cremona C.A., and Behrens A. (2014). ATM signalling and cancer. Oncogene 33, 3351–3360. 10.1038/ONC.2013.275. 23851492

[7] Marabelli M., Cheng S.C., and Parmigiani G. (2016). Penetrance of ATM Gene Mutations in Breast Cancer: A Meta-Analysis of Different Measures of Risk. Genet Epidemiol 40, 425–431. 10.1002/GEPI.21971. 27112364 PMC7376952

[8] Fan X., Wynn J., Shang N., Liu C., Fedotov A., Hallquist M.L.G., Buchanan A.H., Williams M.S., Smith M.E., Hoell C., (2021). Penetrance of Breast Cancer Susceptibility Genes From the eMERGE III Network. JNCI Cancer Spectr 5. 10.1093/JNCICS/PKAB044. PMC8346699 34377931

[9] Hsu F.C., Roberts N.J., Childs E., Porter N., Rabe K.G., Borgida A., Ukaegbu C., Goggins M.G., Hruban R.H., Zogopoulos G., (2021). Risk of Pancreatic Cancer Among Individuals With Pathogenic Variants in the ATM Gene. JAMA Oncol 7, 1664–1668. 10.1001/JAMAONCOL.2021.3701. 34529012 PMC8446906

[10] Dorling L., Carvalho S., Allen J., González-Neira A., Luccarini C., Wahlström C., Pooley K.A., Parsons M.T., Fortuno C., Wang Q., (2021). Breast Cancer Risk Genes - Association Analysis in More than 113,000 Women. N Engl J Med 384, 428–439. 10.1056/NEJMOA1913948. 33471991 PMC7611105

[11] Hu C., Hart S.N., Gnanaolivu R., Huang H., Lee K.Y., Na J., Gao C., Lilyquist J., Yadav S., Boddicker N.J., (2021). A Population-Based Study of Genes Previously Implicated in Breast Cancer. N Engl J Med 384, 440–451. 10.1056/NEJMOA2005936. 33471974 PMC8127622

[12] Veenhuis S., van Os N., Weemaes C., Kamsteeg E.-J., Willemsen M., Adam M.P., Feldman J., Mirzaa G.M., Pagon R.A., Wallace S.E., (2023). Ataxia-Telangiectasia. GeneReviews.

[13] olsen J.H., Hahnemann J.M., Børresen-Dale A.L., Brøndum-Nielsen K., Hammarstrom L., Kleinerman R., Kääriäinen H., Lönnqvist T., Sankila R., Seersholm N., (2001). Cancer in patients with ataxia-telangiectasia and in their relatives in the nordic countries. J Natl Cancer Inst 93, 121–127. 10.1093/JNCI/93.2.121. 11208881

[14] Ahmed M., and Rahman N. (2006). ATM and breast cancer susceptibility. Oncogene 25, 5906–5911. 10.1038/SJ.ONC.1209873. 16998505

[15] Thompson D., Duedal S., Kirner J., McGuffog L., Last J., Reiman A., Byrd P., Taylor M., and Easton D.F. (2005). Cancer risks and mortality in heterozygous ATM mutation carriers. J Natl Cancer Inst 97, 813–822. 10.1093/JNCI/DJI141. 15928302

[16] Tavtigian S. V., Greenblatt M.S., Harrison S.M., Nussbaum R.L., Prabhu S.A., Boucher K.M., and Biesecker L.G. (2018). Modeling the ACMG/AMP Variant Classification Guidelines as a Bayesian Classification Framework. Genet Med 20, 1054. 10.1038/GIM.2017.210. 29300386 PMC6336098

[17] Belman S., Parsons M.T., Spurdle A.B., Goldgar D.E., and Feng B.J. (2020). Considerations in assessing germline variant pathogenicity using cosegregation analysis. Genet Med 22, 2052–2059. 10.1038/S41436-020-0920-4. 32773770

[18] Agaoglu N.B., Bychkovsky B.L., Horton C., Lo M.-T., Polfus L., Carraway C., Hemyari P., Young C., Richardson M.E., Scheib R., (2024). Cancer burden in individuals with single versus double pathogenic variants in cancer susceptibility genes. Genetics in Medicine open 2, 101829. 10.1016/J.GIMO.2024.101829. PMC11613565 39669588

[19] Biesecker L.G., and Harrison S.M. (2018). The ACMG/AMP reputable source criteria for the interpretation of sequence variants. Genet Med 20, 1687–1688. 10.1038/GIM.2018.42. 29543229 PMC6709533

[20] Whiffin N., Minikel E., Walsh R., O’Donnell-Luria A.H., Karczewski K., Ing A.Y., Barton P.J.R., Funke B., Cook S.A., Macarthur D., (2017). Using high-resolution variant frequencies to empower clinical genome interpretation. Genet Med 19, 1151–1158. 10.1038/GIM.2017.26. 28518168 PMC5563454

[21] Swift M., Morrell D., Massey R.B., and Chase C.L. (1991). Incidence of cancer in 161 families affected by ataxia-telangiectasia. N Engl J Med 325, 1831–1836. 10.1056/NEJM199112263252602. 1961222

[22] Swift M, Morrell D, Cromartie E, Chamberlin AR, Skolnick MH, and Bishop DT (1986). The incidence and gene frequency of ataxia-telangiectasia in the United States. Am J Hum Genet 39, 573–583. 3788973 PMC1684065

[23] Zortea M., Armani M., Pastorello E., Nunez G.F., Lombardi S., Tonello S., Rigoni M.T., Zuliani L., Mostacciuolo M.L., Gellera C., (2004). Prevalence of inherited ataxias in the province of Padua, Italy. Neuroepidemiology 23, 275–280. 10.1159/000080092. 15297793

[24] Anheim M., Fleury M., Monga B., Laugel V., Chaigne D., Rodier G., Ginglinger E., Boulay C., Courtois S., Drouot N., (2010). Epidemiological, clinical, paraclinical and molecular study of a cohort of 102 patients affected with autosomal recessive progressive cerebellar ataxia from Alsace, Eastern France: implications for clinical management. Neurogenetics 11, 1–12. 10.1007/S10048-009-0196-Y. 19440741

[25] Anheim M., Fleury M., Monga B., Laugel V., Chaigne D., Rodier G., Ginglinger E., Boulay C., Courtois S., Drouot N., (2010). Epidemiological, clinical, paraclinical and molecular study of a cohort of 102 patients affected with autosomal recessive progressive cerebellar ataxia from Alsace, Eastern France: implications for clinical management. Neurogenetics 11, 1–12. 10.1007/S10048-009-0196-Y. 19440741

[26] Gudmundsson S., Singer-Berk M., Watts N.A., Phu W., Goodrich J.K., Solomonson M., Rehm H.L., MacArthur D.G., and O’Donnell-Luria A. (2022). Variant interpretation using population databases: Lessons from gnomAD. Hum Mutat 43, 1012. 10.1002/HUMU.24309. 34859531 PMC9160216

[27] Southey M.C., Goldgar D.E., Winqvist R., Pylkäs K., Couch F., Tischkowitz M., Foulkes W.D., Dennis J., Michailidou K., van Rensburg E.J., (2016). PALB2, CHEK2 and ATM rare variants and cancer risk: data from COGS. J Med Genet 53, 800–811. 10.1136/JMEDGENET-2016-103839. 27595995 PMC5200636

[28] Thompson D., Duedal S., Kirner J., McGuffog L., Last J., Reiman A., Byrd P., Taylor M., and Easton D.F. (2005). Cancer risks and mortality in heterozygous ATM mutation carriers. J Natl Cancer Inst 97, 813–822. 10.1093/JNCI/DJI141. 15928302

[29] Abou Tayoun A.N., Pesaran T., DiStefano M.T., Oza A., Rehm H.L., Biesecker L.G., and Harrison S.M. (2018). Recommendations for interpreting the loss of function PVS1 ACMG/AMP variant criterion. Hum Mutat 39, 1517–1524. 10.1002/HUMU.23626. 30192042 PMC6185798

[30] Laake K., Jansen L., Hahnemann J., Brondum-Nielsen K., Lonnqvist T., Kaariainen H., Sankila R., Lahdesmaki A., Hammarstrom L., Yuen J., (2000). Characterization of ATM mutations in 41 Nordic families with ataxia telangiectasia. Hum Mutat 16, 232–246. 10980530 10.1002/1098-1004(200009)16:3<232::AID-HUMU6>3.0.CO;2-L

[31] Vořechovský I., Luo L., Prudente S., Chessa L., Russo G., Kanariou M., James M., Negrini M., Webster A.D.B., and Haminarström L. (1996). Exon-scanning mutation analysis of the ATM gene in patients with ataxia-telangiectasia. Eur J Hum Genet 4, 352–355. 10.1159/000472231. 9043869

[32] Teraoka S.N., Telatar M., Becker-Catania S., Liang T., Önengüt S., Tolun A., Chessa L., Sanal Ö., Bernatowska E., Gatti R.A., (1999). Splicing defects in the ataxia-telangiectasia gene, ATM: underlying mutations and consequences. Am J Hum Genet 64, 1617–1631. 10.1086/302418. 10330348 PMC1377904

[33] Landrith T., Li B., Cass A.A., Conner B.R., LaDuca H., McKenna D.B., Maxwell K.N., Domchek S., Morman N.A., Heinlen C., (2020). Splicing profile by capture RNA-seq identifies pathogenic germline variants in tumor suppressor genes. NPJ Precis Oncol 4. 10.1038/S41698-020-0109-Y. PMC7039900 32133419

[34] Stakyte K., Rotheneder M., Lammens K., Bartho J.D., Grädler U., Fuchß T., Pehl U., Alt A., van de Logt E., and Hopfner K.P. (2021). Molecular basis of human ATM kinase inhibition. Nat Struct Mol Biol 28, 789–798. 10.1038/S41594-021-00654-X. 34556870

[35] Baretic D., Pollard H.K., Fisher D.I., Johnson C.M., Santhanam B., Truman C.M., Kouba T., Fersht A.R., Phillips C., and Williams R.L. (2017). Structures of closed and open conformations of dimeric human ATM. Sci Adv 3. 10.1126/SCIADV.1700933. PMC5425235 28508083

[36] Yates L.A., Williams R.M., Hailemariam S., Ayala R., Burgers P., and Zhang X. (2020). Cryo-EM Structure of Nucleotide-Bound Tel1ATM Unravels the Molecular Basis of Inhibition and Structural Rationale for Disease-Associated Mutations. Structure 28, 96–104.e3. 10.1016/J.STR.2019.10.012. 31740029 PMC6945111

[37] Xiao J., Liu M., Qi Y., Chaban Y., Gao C., Pan B., Tian Y., Yu Z., Li J., Zhang P., (2019). Structural insights into the activation of ATM kinase. Cell Res 29, 683–685. 10.1038/S41422-019-0205-0. 31320732 PMC6796860

[38] Lee J.H., and Paull T.T. (2007). Activation and regulation of ATM kinase activity in response to DNA double-strand breaks. Oncogene 26, 7741–7748. 10.1038/SJ.ONC.1210872. 18066086

[39] Verhagen M.M.M., Abdo W.F., Willemsen M.A.A.P., Hogervorst F.B.L., Smeets D.F.C.M., Hiel J.A.P., Brunt E.R., Van Rijn M.A., Majoor Krakauer D., Oldenburg R.A., (2009). Clinical spectrum of ataxia-telangiectasia in adulthood. Neurology 73, 430–437. 10.1212/WNL.0B013E3181AF33BD. 19535770

[40] Van Os N.J.H., Chessa L., Weemaes C.M.R., Van Deuren M., Fiévet A., Van Gaalen J., Mahlaoui N., Roeleveld N., Schrader C., Schindler D., (2019). Genotype-phenotype correlations in ataxia telangiectasia patients with ATM c.3576G>A and c.8147T>C mutations. J Med Genet 56, 308–316. 10.1136/JMEDGENET-2018-105635. 30819809

[41] Dörk T., Bendix-Waltes R., Wegner R.D., and Stumm M. (2004). Slow progression of ataxia-telangiectasia with double missense and in frame splice mutations. Am J Med Genet A 126A, 272–277. 10.1002/AJMG.A.20601. 15054841

[42] Zannolli R., Sabrina Buoni, Betti G., Salvucci S., Plebani A., Soresina A., Pietrogrande M.C., Martino S., Leuzzi V., Finocchi A., (2012). A randomized trial of oral betamethasone to reduce ataxia symptoms in ataxia telangiectasia. Mov Disord 27, 1312–1316. 10.1002/MDS.25126. 22927201

[43] Chessa L., Petrinelli P., Antonelli A., Fiorilli M., Elli R., Marcucci L., Federico A., and Gandini E. (1992). Heterogeneity in ataxia-telangiectasia: classical phenotype associated with intermediate cellular radiosensitivity. Am J Med Genet 42, 741–746. 10.1002/AJMG.1320420524. 1632451

[44] Magliozzi M., Piane M., Torrente I., Sinibaldi L., Rizzo G., Savio C., Lulli P., De Luca A., Dallapiccola B., and Chessa L. (2006). DHPLC screening of ATM gene in Italian patients affected by ataxia-telangiectasia: fourteen novel ATM mutations. Dis Markers 22, 257–264. 10.1155/2006/740493. 17124347 PMC3862285

[45] Gilad S., Khosravi R., Shkedy D., Uziel T., Ziv Y., Savitsky K., Rotman G., Smith S., Chessa L., Jorgensen T.J., (1996). Predominance of null mutations in ataxia-telangiectasia. Hum Mol Genet 5, 433–439. 10.1093/HMG/5.4.433. 8845835

[46] Mitui M., Bernatowska E., Pietrucha B., Piotrowska-Jastrzebska J., Eng L., Nahas S., Teraoka S., Sholty G., Purayidom A., Concannon P., (2005). ATM gene founder haplotypes and associated mutations in Polish families with ataxia-telangiectasia. Ann Hum Genet 69, 657–664. 10.1111/J.1529-8817.2005.00199.X. 16266405

[47] Stankovic T., Kidd A.M.J., Sutcliffe A., McGuire G.M., Robinson P., Weber P., Bedenham T., Bradwell A.R., Easton D.F., Lennox G.G., (1998). ATM mutations and phenotypes in ataxia-telangiectasia families in the British Isles: expression of mutant ATM and the risk of leukemia, lymphoma, and breast cancer. Am J Hum Genet 62, 334–345. 10.1086/301706. 9463314 PMC1376883

[48] Davis M.Y., Keene C.D., Swanson P.D., Sheehy C., and Bird T.D. (2013). Novel mutations in ataxia telangiectasia and AOA2 associated with prolonged survival. J Neurol Sci 335, 134–138. 10.1016/J.JNS.2013.09.014. 24090759 PMC4017341

[49] Verhagen M.M.M., Last J.I., Hogervorst F.B.L., Smeets D.F.C.M., Roeleveld N., Verheijen F., Catsman-Berrevoets C.E., Wulffraat N.M., Cobben J.M., Hiel J., (2012). Presence of ATM protein and residual kinase activity correlates with the phenotype in ataxia-telangiectasia: a genotype-phenotype study. Hum Mutat 33, 561–571. 10.1002/HUMU.22016. 22213089

[50] Buzin C.H., Gatti R.A., Nguyen V.Q., Wen C.Y., Mitui M., Sanal O., Chen J.S., Nozari G., Mengos A., Li X., (2003). Comprehensive scanning of the ATM gene with DOVAM-S. Hum Mutat 21, 123–131. 10.1002/HUMU.10158. 12552559

[51] Schon K., van Os N.J.H., Oscroft N., Baxendale H., Scoffings D., Ray J., Suri M., Whitehouse W.P., Mehta P.R., Everett N., (2019). Genotype, extrapyramidal features, and severity of variant ataxia-telangiectasia. Ann Neurol 85, 170–180. 10.1002/ANA.25394. 30549301 PMC6590299

[52] Byrd P.J., Srinivasan V., Last J.I., Smith A., Biggs P., Carney E.F., Exley A., Abson C., Stewart G.S., Izatt L., (2012). Severe reaction to radiotherapy for breast cancer as the presenting feature of ataxia telangiectasia. Br J Cancer 106, 262–268. 10.1038/BJC.2011.534. 22146522 PMC3261689

[53] Reiman A., Srinivasan V., Barone G., Last J.I., Wootton L.L., Davies E.G., Verhagen M.M., Willemsen M.A., Weemaes C.M., Byrd P.J., (2011). Lymphoid tumours and breast cancer in ataxia telangiectasia; substantial protective effect of residual ATM kinase activity against childhood tumours. Br J Cancer 105, 586–591. 10.1038/BJC.2011.266. 21792198 PMC3170966

[54] Sandoval N., Platzer M., Rosenthal A., Dörk T., Bendix R., Skawran B., Stuhrmann M., Wegner R.D., Sperling K., Banin S., (1999). Characterization of ATM gene mutations in 66 ataxia telangiectasia families. Hum Mol Genet 8, 69–79. 10.1093/HMG/8.1.69. 9887333

[55] Isik E., Onay H., Atik T., Canda E., Cogulu O., Coker M., and Ozkinay F., (2019). Clinical utility of a targeted next generation sequencing panel in severe and pediatric onset Mendelian diseases. Eur J Med Genet 62. 10.1016/J.EJMG.2019.103725. 31319225

[56] Cavalieri S., Funaro A., Pappi P., Migone N., Gatti R.A., and Brusco A. (2008). Large genomic mutations within the ATM gene detected by MLPA, including a duplication of 41 kb from exon 4 to 20. Ann Hum Genet 72, 10–18. 10.1111/J.1469-1809.2007.00399.X. 17910737

[57] Carney E.F., Srinivasan V., Moss P.A., and Taylor A.M. (2012). Classical ataxia telangiectasia patients have a congenitally aged immune system with high expression of CD95. J Immunol 189, 261–268. 10.4049/JIMMUNOL.1101909. 22649200

[58] Liu X.L., Wang T., Huang X.J., Zhou H.Y., Luan X.H., Shen J.Y., Chen S. Di, and Cao L. (2016). Novel ATM mutations with ataxia-telangiectasia. Neurosci Lett 611, 112–115. 10.1016/J.NEULET.2015.11.036. 26628246

[59] Chessa L., Piane M., Magliozzi M., Torrente I., Savio C., Lulli P., De Luca A., and Dallapiccola B. (2009). Founder effects for ATM gene mutations in Italian Ataxia Telangiectasia families. Ann Hum Genet 73, 532–539. 10.1111/J.1469-1809.2009.00535.X. 19691550

[60] McConville C.M., Stankovic T., Byrd P.J., McGuire G.M., Yao Q.Y., Lennox G.G., and Taylor A.M.R. (1996). Mutations associated with variant phenotypes in ataxia-telangiectasia. Am J Hum Genet 59, 320. 8755918 PMC1914715

[61] Toyoshima M., Hara T., Zhang H., Yamamoto T., Akaboshi S., Nanba E., Ohno K., Hori N., Sato K., and Takeshita K., (1998). Ataxia-telangiectasia without immunodeficiency: novel point mutations within and adjacent to the phosphatidylinositol 3-kinase-like domain - PubMed. Am J Hum Genet, 141–144. 9450874

[62] Byrd P.J., Srinivasan V., Last J.I., Smith A., Biggs P., Carney E.F., Exley A., Abson C., Stewart G.S., Izatt L., (2012). Severe reaction to radiotherapy for breast cancer as the presenting feature of ataxia telangiectasia. Br J Cancer 106, 262–268. 10.1038/BJC.2011.534. 22146522 PMC3261689

[63] Lin J.H., Tang X.Y., Boulling A., Zou W. Bin, Masson E., Fichou Y., Raud L., Le Tertre M., Deng S.J., Berlivet I., (2019). First estimate of the scale of canonical 5’ splice site GT>GC variants capable of generating wild-type transcripts. Hum Mutat 40, 1856–1873. 10.1002/HUMU.23821. 31131953

[64] Walker L.C., Hoya M. de la, Wiggins G.A.R., Lindy A., Vincent L.M., Parsons M.T., Canson D.M., Bis-Brewer D., Cass A., Tchourbanov A., (2023). Using the ACMG/AMP framework to capture evidence related to predicted and observed impact on splicing: Recommendations from the ClinGen SVI Splicing Subgroup. Am J Hum Genet. 10.1016/J.AJHG.2023.06.002. PMC10357475 37352859

[65] Ioannidis N.M., Rothstein J.H., Pejaver V., Middha S., McDonnell S.K., Baheti S., Musolf A., Li Q., Holzinger E., Karyadi D., (2016). REVEL: An Ensemble Method for Predicting the Pathogenicity of Rare Missense Variants. Am J Hum Genet 99, 877–885. 10.1016/J.AJHG.2016.08.016. 27666373 PMC5065685

[66] Choi Y., and Chan A.P. (2015). PROVEAN web server: a tool to predict the functional effect of amino acid substitutions and indels. Bioinformatics 31, 2745. 10.1093/BIOINFORMATICS/BTV195. 25851949 PMC4528627

[67] Choi Y., Sims G.E., Murphy S., Miller J.R., and Chan A.P. (2012). Predicting the functional effect of amino acid substitutions and indels. PLoS One 7. 10.1371/JOURNAL.PONE.0046688. PMC3466303 23056405

[68] Cubuk C., Garrett A., Choi S., King L., Loveday C., Torr B., Burghel G.J., Durkie M., Callaway A., Robinson R., (2021). Clinical likelihood ratios and balanced accuracy for 44 in silico tools against multiple large-scale functional assays of cancer susceptibility genes. Genet Med 23, 2096–2104. 10.1038/S41436-021-01265-Z. 34230640 PMC8553612

[69] Jaganathan K., Kyriazopoulou Panagiotopoulou S., McRae J.F., Darbandi S.F., Knowles D., Li Y.I., Kosmicki J.A., Arbelaez J., Cui W., Schwartz G.B., (2019). Predicting Splicing from Primary Sequence with Deep Learning. Cell 176, 535–548.e24. 10.1016/J.CELL.2018.12.015. 30661751

[70] Scott S.P., Bendix R., Chen P., Clark R., Dörk T., and Lavin M.F. (2002). Missense mutations but not allelic variants alter the function of ATM by dominant interference in patients with breast cancer. Proc Natl Acad Sci U S A 99, 925–930. 10.1073/PNAS.012329699. 11805335 PMC117407

[71] Barone G., Groom A., Reiman A., Srinivasan V., Byrd P.J., and Taylor A.M.R. (2009). Modeling ATM mutant proteins from missense changes confirms retained kinase activity. Hum Mutat 30, 1222–1230. 10.1002/HUMU.21034. 19431188

[72] Brnich S.E., Abou Tayoun A.N., Couch F.J., Cutting G.R., Greenblatt M.S., Heinen C.D., Kanavy D.M., Luo X., McNulty S.M., Starita L.M., (2019). Recommendations for application of the functional evidence PS3/BS3 criterion using the ACMG/AMP sequence variant interpretation framework. Genome Med 12. 10.1186/S13073-019-0690-2. PMC6938631 31892348

[73] Saunders-Pullman R., Raymond D., Stoessl A.J., Hobson D., Nakamura T., Pullman S., Lefton D., Okun M.S., Uitti R., Sachdev R., (2012). Variant ataxia-telangiectasia presenting as primary-appearing dystonia in Canadian Mennonites. Neurology 78, 649–657. 10.1212/WNL.0B013E3182494D51. 22345219 PMC3286230

[74] Feliubadaló L., Moles-Fernández A., Santamariña-Pena M., Sánchez A.T., López-Novo A., Porras L.M., Blanco A., Capellá G., de la Hoya M., Molina I.J., (2021). A Collaborative Effort to Define Classification Criteria for ATM Variants in Hereditary Cancer Patients. Clin Chem 67, 518–533. 10.1093/CLINCHEM/HVAA250. 33280026

