# Supplementary material for: Specifications of the ACMG/AMP variant curation guidelines for the analysis of germline ATM sequence variants
Source: medRxiv. 2024 May 29:2024.05.28.24307502. Preprint. [Version 1] doi: 10.1101/2024.05.28.24307502 (PMC11160822; doi:10.1101/2024.05.28.24307502)
Supplement: Supplement 1 [file NIHPP2024.05.28.24307502v1-supplement-1.pdf]

Supplementary Figure 1

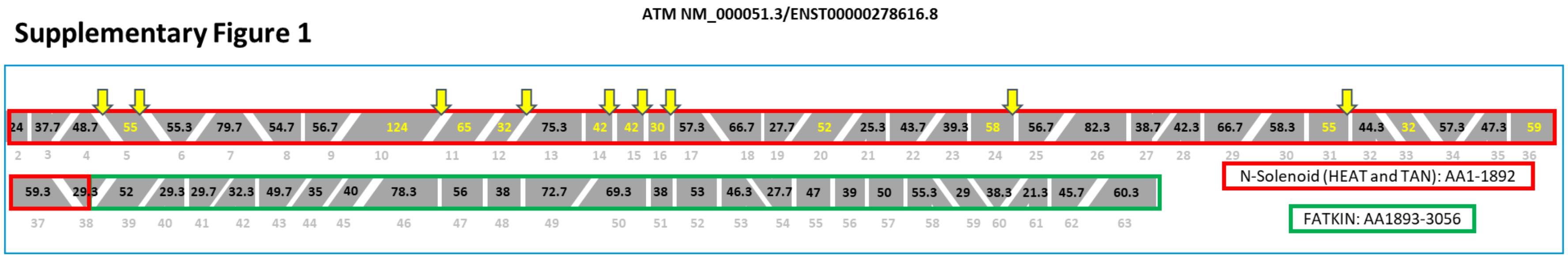

Supplementary Figure 1. In-Frame Losses in the N-Solenoid Domain Cause A-T. *A) The ATM gene depicted exon-by-exon as in Figure 2 (gray text on bottom is total exon numbering) with in frame exons within the N-Solenoid domain indicated by yellow text. Yellow down-facing arrows indicate a variant identified at that splice junction in a patient with A-T. Details of the observed in-frame splice defect, patient genotype and citation are described in the table.*

Supplementary Table 1.

Supplementary Table 1. N-Solenoid losses for A-T Patients

Site c. (Exon)	Observed transcript (p.)	# AA lost	Ataxia-Telangiectasia Patients
c.332 (Exon 5)	r.332_496del (p.Arg111_Glu166delinsK)[1]	56	ATM c.332-1G>A CH with ATM c.2250G>A[1]
c.496 (Exon 5)	r.332_496del (p.Arg111_Glu166delinsK)[2]	56	ATM c.496+5G>A CH with ATM c.7875_7876delTGinsGC[2-4]
c.1607 (Exon 10)	r.1407_1607del (p.Arg469_Cys536delinsSer)[7]	67	ATM c.1607+1G>T homozygous and CH with ATM c.3576G>A (p.K1192K) or variant not identified[5-8]
	Intron retention also reported[5]		
c.1898 (Exon 12)	r.1803_1898del (p.Asn602_Cys633del)[9]	32	ATM c.1898+1G>T CH with ATM EX21_29del[9]; ATM c.1898+2T>G CH with ATM c.5825C>T (p.A1942V), c.3085_6086insA, or unidentified 2nd variant[10-12]
c.2250 (Exon 14)	r.2125_2250del (p.Ile709_Lys750del) (Ambry, internal data)	42	ATM c.332-1G>A CH with ATM c.2250G>A[1]
c.2376 (Exon 15)	r.2251_2376del (p.Ser751_Lys792del) (Ambry, internal data)	42	ATM c.2376+1G>A CH with ATM c.7875_7876delTGinsGC[13]
c.2466 (Exon 16)	r.2377_2466del (p.Lys793_Leu822del) (Ambry, internal data)	30	ATM c.2466+1delG CH with ATM c.6913C>T (p.Q2305*) or unidentified 2nd variant[5, 9, 14]

c.3576 (Exon 24)	r.3403_3576del (p.Ser1135_K1192del)[17,18]	58	ATM c.3576G>A CH with c.3111delT, c.2413C>T (p.Arg805*), c.1369C>T (p.Arg457*), c.4842_4843insCT, c.8545C>T (p.Arg2849*); c.1607+1G>T; c.6761A>C (p.His2254Pro))[5,9,15,16,17]; ATM c.3576G>A homozygous (2 patients)[7,13,15,18]
c.4776 (Exon 31)	r.4612_4776del165 (p.V1538_E1592del)[20]	55	ATM c.4776+1G>T CH with ATM c.748C>T (p.Arg250*); c.8977C>T (p.Arg2993*); or unknown variant[11,19,20]; ATM c.4776+2T>C homozygote[5,7]
CH = Compound Heterozygous			

Supplementary Table 1 References

1. Laake, K., Jansen, L., Hahnemann, J., Brondum-Nielsen, K., Lonnqvist, T., Kaariainen, H., Sankila, R., Lahdesmaki, A., Hammarstrom, L., Yuen, J., et al. (2000). Characterization of ATM mutations in 41 Nordic families with ataxia telangiectasia. Hum Mutat 16, 232–246.

2. Dörk, T., Bendix-Waltes, R., Wegner, R.D., and Stumm, M. (2004). Slow progression of ataxia-telangiectasia with double missense and in frame splice mutations. Am J Med Genet A 126A, 272–277. 10.1002/AJMG.A.20601.

3. Van Os, N.J.H., Chessa, L., Weemaes, C.M.R., Van Deuren, M., Fiévet, A., Van Gaalen, J., Mahlaoui, N., Roeleveld, N., Schrader, C., Schindler, D., et al. (2019). Genotype-phenotype correlations in ataxia telangiectasia patients with ATM c.3576G>A and c.8147T>C mutations. J Med Genet 56, 308–316. 10.1136/JMEDGENET-2018-105635.
4. Verhagen, M.M.M., Abdo, W.F., Willemsen, M.A.A.P., Hogervorst, F.B.L., Smeets, D.F.C.M., Hiel, J.A.P., Brunt, E.R., Van Rijn, M.A., Majoor Krakauer, D., Oldenburg, R.A., et al. (2009). Clinical spectrum of ataxia-telangiectasia in adulthood. Neurology 73, 430–437. 10.1212/WNL.0B013E3181AF33BD.
5. Teraoka, S.N., Telatar, M., Becker-Catania, S., Liang, T., Önengüt, S., Tolun, A., Chessa, L., Sanal, Ö., Bernatowska, E., Gatti, R.A., et al. (1999). Splicing defects in the ataxia-telangiectasia gene, ATM: underlying mutations and consequences. Am J Hum Genet 64, 1617–1631. 10.1086/302418.
6. Magliozzi, M., Piane, M., Torrente, I., Sinibaldi, L., Rizzo, G., Savio, C., Lulli, P., De Luca, A., Dallapiccola, B., and Chessa, L. (2006). DHPLC screening of ATM gene in Italian patients affected by ataxia-telangiectasia: fourteen novel ATM mutations. Dis Markers 22, 257–264. 10.1155/2006/740493.
7. Gilad, S., Khosravi, R., Shkedy, D., Uziel, T., Ziv, Y., Savitsky, K., Rotman, G., Smith, S., Chessa, L., Jorgensen, T.J., et al. (1996). Predominance of null mutations in ataxia-telangiectasia. Hum Mol Genet 5, 433–439. 10.1093/HMG/5.4.433.

8. Telatar, M., Teraoka, S., Wang, Z., Chun, H.H., Liang, T., Castellvi-Bel, S., Udar, N., Borresen-Dale, A.L., Chessa, L., Bernatowska-Matuszkiewicz, E., et al. (1998). Ataxia-telangiectasia: identification and detection of founder-effect mutations in the ATM gene in ethnic populations. Am J Hum Genet 62, 86–97. 10.1086/301673.
9. Cavalieri, S., Funaro, A., Pappi, P., Migone, N., Gatti, R.A., and Brusco, A. (2008). Large genomic mutations within the ATM gene detected by MLPA, including a duplication of 41 kb from exon 4 to 20. Ann Hum Genet 72, 10–18. 10.1111/J.1469-1809.2007.00399.X.

10. Davis, M.Y., Keene, C.D., Swanson, P.D., Sheehy, C., and Bird, T.D. (2013). Novel mutations in ataxia telangiectasia and AOA2 associated with prolonged survival. J Neurol Sci 335, 134–138. 10.1016/J.JNS.2013.09.014.

11. Mitui, M., Bernatowska, E., Pietrucha, B., Piotrowska-Jastrzebska, J., Eng, L., Nahas, S., Teraoka, S., Sholty, G., Purayidom, A., Concannon, P., et al. (2005). ATM gene founder haplotypes and associated mutations in Polish families with ataxia-telangiectasia. Ann Hum Genet 69, 657–664. 10.1111/J.1529-8817.2005.00199.X.
12. Stankovic, T., Kidd, A.M.J., Sutcliffe, A., McGuire, G.M., Robinson, P., Weber, P., Bedenham, T., Bradwell, A.R., Easton, D.F., Lennox, G.G., et al. (1998). ATM mutations and phenotypes in ataxia-telangiectasia families in the British Isles: expression of mutant ATM and the risk of leukemia, lymphoma, and breast cancer. Am J Hum Genet 62, 334–345. 10.1086/301706.
13. Verhagen, M.M.M., Last, J.I., Hogervorst, F.B.L., Smeets, D.F.C.M., Roeleveld, N., Verheijen, F., Catsman-Berrevoets, C.E., Wulffraat, N.M., Cobben, J.M., Hiel, J., et al. (2012). Presence of ATM protein and residual kinase activity correlates with the phenotype in ataxia-telangiectasia: a genotype-phenotype study. Hum Mutat 33, 561–571. 10.1002/HUMU.22016.
14. Zannolli, R., Sabrina Buoni, Betti, G., Salvucci, S., Plebani, A., Soresina, A., Pietrogrande, M.C., Martino, S., Leuzzi, V., Finocchi, A., et al. (2012). A randomized trial of oral betamethasone to reduce ataxia symptoms in ataxia telangiectasia. Mov Disord 27, 1312–1316. 10.1002/MDS.25126.
15. Chessa, L., Piane, M., Magliozzi, M., Torrente, I., Savio, C., Lulli, P., De Luca, A., and Dallapiccola, B. (2009). Founder effects for ATM gene mutations in Italian Ataxia Telangiectasia families. Ann Hum Genet 73, 532–539. 10.1111/J.1469-1809.2009.00535.X.
16. Jacquemin, V., Rieunier, G., Jacob, S., Bellanger, D., D'Enghien, C.D., Laugé, A., Stoppa-Lyonnet, D., and Stern, M.H. (2012). Underexpression and abnormal localization of ATM products in ataxia telangiectasia patients bearing ATM missense mutations. Eur J Hum Genet 20, 305–312. 10.1038/EJHG.2011.196.
17. Sandoval, N., Platzer, M., Rosenthal, A., Dörk, T., Bendix, R., Skawran, B., Stuhrmann, M., Wegner, R.D., Sperling, K., Banin, S., et al. (1999). Characterization of ATM gene mutations in 66 ataxia telangiectasia families. Hum Mol Genet 8, 69–79. 10.1093/HMG/8.1.69.
18. Demuth, I., Dutrannoy, V., Marques, W., Neitzel, H., Schindler, D., Dimova, P.S., Chrzanowska, K.H., Bojinova, V., Gregorek, H., Graul-Neumann, L.M., et al. (2011). New mutations in the ATM gene and clinical data of 25 AT patients. Neurogenetics 12, 273–282. 10.1007/S10048-011-0299-0.
19. Broccoletti, T., Del Giudice, E., Cirillo, E., Vigliano, I., Giardino, G., Ginocchio, V.M., Bruscoli, S., Riccardi, C., and Pignata, C. (2011). Efficacy of very-low-dose betamethasone on neurological symptoms in ataxia-telangiectasia. Eur J Neurol 18, 564–570. 10.1111/J.1468-1331.2010.03203.X.
20. Fiévet, A., Bellanger, D., Rieunier, G., Dubois d'Enghien, C., Sophie, J., Calvas, P., Carriere, J.P., Anheim, M., Castrioto, A., Flabeau, O., et al. (2019). Functional classification of ATM variants in ataxia-telangiectasia patients. Hum Mutat 40, 1713–1730. 10.1002/HUMU.23778.

Supplementary Table 2. **SpATM-WG vs HBOP Classification Criteria Comparison.**

		Spanish ATM Working Group (SpATM-WG)	ClinGen HBOP VCEP	Difference	Rationale
Population	BA1-Population Frequency Stand Alone	>.5%	>.5% per filtering allele frequency	HBOP more conservative	HBOP requires statistical models be applied to the frequency before applying
	BS1-Population Frequency Strong	>.05%	>.05% per filtering allele frequency		
	PM2-Rarity	<.001% in total gnomAD or <.002% in sub-population gnomAD if N≥2	<.001% in all sub-populations Singletons receive PM2_Supporting regardless of allele frequency	HBOP more conservative	HBOP has a lower threshold for sub-populations to ascribe PM2_Supporting
Computational/Predictive	BP1-Missense in a LoF Gene	N/A	N/A	No difference	
	BP3-in frame indel in a repeat region	N/A	N/A	No difference	
	BP4-in silico benign	VEST4 & REVEL <.5 (domain restricted) SPiCE 2.1: <0.240 (donor) <0.282 (acceptor)	REVEL <.249 SpliceAI ≤.1	HBOP less conservative	HBOP requires only one metapredictor and does not restric its use by domain or position.
	PP3-in silico deleterious	VEST4 & REVEL >.5 (domain restricted) SPiCE 2.1: ≥0.240 (donor) ≥0.789 (acceptor)	REVEL >.733 SpliceAI ≥.2		
	BP5-different-gene co-occurrences	N/A	N/A	No difference	
	BP7-synonymous/deep intronic	Unconserved synonymous	Synonymous and deep-intronic	HBOP ignores conservation and includes deep-intronic per SVI guidelines	
	PP2-missense constraint	N/A	N/A	No difference	
	PM4-in frame indel and stop-loss	Applies: codon and domain-specific	Use for stop-loss only	HBOP more conservative	HBOP does not allow PM4 for in frame events due to inability to accurately predict which are pathogenic and which are neutral
	PM5-missense hotspot	Applies: in silico informed restrictions	Does not apply as hotspot. Co-opted code (as supporting) for truncations upstream of p.Arg3047	HBOP more conservative HBOP less conservative	HBOP does not allow PM5 as a hotspot due to inability to accurartely predict which are pathogenic and which are neutral. SpATM-WG does not use PM5_Supporting as a co-opted code
	PM1-structural domain hospot	Applies: codon and domain-specific	N/A	HBOP more conservative	HBOP did not conduct a similar analysis as SpATM-WG because frequency is not a sole predictor of pathogenicity
	PS1-same AA change as LP/P	Applies for protein. Also used (as supporting) for RNA hotspot	Applies for protein. Also used for RNA hostpot with restrictions per SVI guidelines	HBOP more current	SVI guidelines for RNA were not published at the time of SpATM-WG's publication
Phenotype Data	BS2-Healthy Adult	Used for biallelic healthy patients with restrictions	N/A	Code difference	HBOP codes biallelic healthy patients as BP2
	PVS1-LoF	Reference SVI's Tayoun et al	Detailed Decision Tree Provided	HBOP more current	HBOP also used the Tayoun et al decision tree as a basis but detailed it for ATM as a reference for biocurators.
	PP4-phenotype highly consistent	N/A	N/A	No difference	
	PS4-case control/proband counting	Applies at variable weight: proband counting of A-T families with restrictions.	Case Control Only; no proband counting	Code difference	HBOP codes biallelic affected patients as PM3. SpATM-WG restricts the use of both PM3 and PS4
Functional	BS3-functional benign	Applies to RNA and Protein studies. Protein studies include patient- or cell line studies ascertaining ATM auto- and trans-phosphorylation and sensitivity to DNA damaging agents	Applies to protein studies: Protein studies include ATM-null cell lines ascertaining ATM-specific phosphorylation and/or radiosensitivity. Max weight BS3_Supporting *each.*	HBOP more conservative	Few known-pathogenic and known-benign variants are included in functional studies leading to an inability to validate them at strong per Brnich et al
	PS3-functional pathogenic	Applies to RNA and Protein studies. Protein studies include patient- or cell line studies ascertaining ATM auto- and trans-phosphorylation and sensitivity to DNA damaging agents. Max weight PS3 as strong	Applies to protein studies: Protein studies include ATM-null cell lines ascertaining ATM-specific phosphorylation and radiosensitivity. Max weight PS3_Moderate for both or PS3_Supporting for ATM-specific event only. No weight for radiosensitivity-only.		
	BP7(RNA)-assay with no aberrant splicing	Coded as BS3	Applies per SVI guidelines	HBOP more current	SVI guidelines for RNA were not published at the time of SpATM-WG's publication
	PVS1(RNA)-assay with aberrant splicing	Coded as PS3			

Segregation	**BS4-non segregation**	Applies to A-T families	N/A	Different codes	HBOP would apply unaffected biallelic patients (even if siblings of an A-T patient) as BP2, instead
	**PP1-segregation with disease**	Applies to A-T families	N/A	HBOP more conservative	Segregation within A-T families is extremely rare in the literature and cannot be used to inform the appropriate weighting of such occurrences at this time
de novo	**PM6-presumed *de novo***	Applies to A-T families	N/A	HBOP more conservative	De novo occurrences in the heterozygous state are not informative. Do novo occurrences in the biallelic state would be challenging to determine phase without long-read technologies due to the size of ATM
	**PS2-confirmed *de novo***	Applies to A-T families	N/A		
Allelic	**BP2-biallelic unaffected**	Applies. Unrestricted	Applies with restrictions	HBOP more conservative	Additional precautions are applied around the age of the proband, the phase/zygosity, the diagnostic setting and the variant population frequency
	**PM3-biallelic affected**	Applies per ClinGen SVI Recommendataions PM3 Version 1.0 with restrictions	Applies at double the weight from SVI Recommendations for PM3 Version 1.0. Restrictions for phenotype specificity, phase/zygosity, variant classifications	HBOP is less conservative	There is a 1:1 relationship between the genotype and phenotype from A-T patients. A-T clinical criteria are detailed in HBOP rules
